# tsRNA-3025a Impairs Mitochondrial Function and Autophagy to Inhibit Myocardial Regeneration and Repair Following Ischemia–Reperfusion Injury

**DOI:** 10.3390/jcdd13060266

**Published:** 2026-06-12

**Authors:** Zehao Feng, Xing Li, Ai Zhou, Han Zhang, Kaixuan Tang, Yumo Yang, Ying Chen, Li Zhang, Lingmei Qian

**Affiliations:** 1 Department of Cardiology & Hongqiao International Institute of Medicine, Tongren Hospital, Shanghai Jiao Tong University School of Medicine, Shanghai 200051, China; theofung@sjtu.edu.cn (Z.F.); han_zhang111@126.com (H.Z.); 15288820976@163.com (K.T.); y_yumo@126.com (Y.Y.); 2 Department of Cardiology, Jiangnan University Medical Center (Wuxi No. 2 People’s Hospital), Wuxi School of Medicine, Jiangnan University, Wuxi 214043, China; 9862023140@jiangnan.edu.cn (X.L.); zhouai182516@163.com (A.Z.); 3 Department of Radiation Oncology, Tongren Hospital, Shanghai Jiao Tong University School of Medicine, Shanghai 200051, China; cy1794@shtrhospital.com

**Keywords:** tsRNA, ischemia–reperfusion injury, autophagy, PIK3C2A, cardiomyocyte proliferation

## Abstract

Myocardial ischemia–reperfusion (I/R) injury is a frequent complication of acute myocardial infarction (AMI), yet clinical biomarkers and targets remain limited. Although tRNA-derived small RNAs (tsRNAs) are emerging cardiovascular regulators, their roles in I/R injury are not fully elucidated. We identified tsRNA-3025a via sequencing in mouse I/R models and validated its clinical significance. Circulating tsRNA-3025a was significantly upregulated in AMI and unstable angina patients, independently predicting adverse events within 30 days. Functionally, tsRNA-3025a exacerbated apoptosis and mitochondrial dysfunction in vitro, while its in vivo silencing reduced infarct size, improved cardiac function and increased the proportion of Ki67- and pH3-positive cardiomyocytes. Mechanistically, tsRNA-3025a aggravated injury by targeting PIK3C2A, thereby suppressing autophagosome formation and impairing protective autophagic flux during reperfusion. In conclusion, circulating tsRNA-3025a serves as a prognostic biomarker for post-PCI patients. Targeting tsRNA-3025a attenuates myocardial I/R injury and restores myocardial regeneration and repair by regulating PIK3C2A-mediated protective autophagy flux.

## 1. Introduction

Coronary artery disease (CAD) is one of the major causes of global mortality [1]. Recent studies reveal that CAD-related mortality has reached 123 per 100,000 people in the United States and increased by 46% between 1990 and 2013 in China [2,3]. Acute myocardial infarction (AMI), representing the most critical phenotype of CAD, is mostly triggered by plaque rupture and subsequent thrombosis, demanding urgent revascularization measures like percutaneous coronary intervention (PCI) [4,5]. While PCI significantly reduces mortality, it inevitably induces myocardial ischemia–reperfusion (I/R) injury, a complication that can precipitate ventricular arrhythmias and acute heart failure [4,6]. Furthermore, given the limited proliferative capacity of cardiomyocytes, the massive loss of cardiomyocytes following AMI is eventually replaced by fibrotic scar tissue, leading to irreversible ventricular remodeling. Despite advances in reperfusion therapy, effective predictive biomarkers and therapeutic targets for mitigating I/R injury remain scarce. Thus, elucidating the molecular pathophysiology of I/R injury is critical for improving clinical outcomes.

Non-coding RNAs (ncRNAs) are increasingly recognized as critical modulators of cardiovascular diseases [7,8,9,10,11]. Among them, transfer RNA-derived small RNAs (tsRNAs) represent a novel class of regulatory fragments generated from tRNA cleavage [12]. Unlike random degradation products, tsRNA biogenesis is a precise, enzymatically controlled process. Dictated by their fragment size and specific processing sites, tsRNAs are typically distinguished into two subclasses: tRNA-derived fragments (tRFs) and tRNA-derived stress-induced RNAs (tiRNAs). The larger tiRNAs (30–40 nt), frequently referred to as tRNA halves, are generated via Angiogenin (ANG)-mediated anticodon loop cleavage under conditions of cellular stress [13]. In contrast, the shorter tRFs (14–30 nt) generally stem from the Dicer-mediated processing of mature tRNA 5′- or 3′-termini [14,15]. These fragments are remarkably stable in circulation and can incorporate into Argonaute (Ago) complexes to regulate gene expression post-transcriptionally, acting similarly to microRNAs [16]. Given their specific induction by stress stimuli and high stability, tsRNAs are hypothesized to play a crucial role in the acute cellular response to ischemic stress [17,18].

Recent evidence highlights the functional involvement of tsRNAs in myocardial I/R injury [18,19]. Additionally, previous studies have reported that 5′tiRNA-Gly-CCC promotes skeletal muscle regeneration, suggesting that tsRNAs may also play a role in myocardial regeneration repair [20]. Crucially, tsRNAs have recently been linked to autophagy, a fundamental cellular mechanism for maintaining homeostasis during stress. Deng et al. reported that tiRNA-Met-CAT-002 exacerbates myocardial injury by suppressing Bnip3-mediated autophagy [21]. Autophagy plays a dual role in I/R injury; while moderate autophagy is generally protective by clearing damaged organelles, its dysregulation leads to cardiomyocyte death [22,23]. Autophagy is a tightly regulated process involving multiple signaling nodes, such as the phosphatidylinositol 3-kinase (PI3K) family, which governs autophagosome formation [24]. However, the specific landscape of ischemia-responsive tsRNAs and the precise mechanisms by which they coordinate the complex autophagy machinery remain largely unexplored.

In the present study, we identified a functionally significant tsRNA, tsRNA-3025a, which is markedly upregulated in both unstable angina (UA) and AMI patients and correlates with adverse clinical outcomes. Functionally, we demonstrate that tsRNA-3025a acts as a pathogenic driver of I/R injury by exacerbating cardiomyocyte apoptosis, mitochondrial dysfunction, and adverse cardiac remodeling. Mechanistically, we further reveal that tsRNA-3025a suppresses protective autophagy flux by directly targeting PIK3C2A. Collectively, our findings establish tsRNA-3025a as both a prognostic biomarker in AMI patients and a promising therapeutic target for I/R injury.

## 2. Materials and Methods

### 2.1. Study Design and Participants

We conducted a two-center observational cohort study to evaluate the predictive value of tsRNA-3025a in patients with acute coronary syndrome (ACS) (Trial Registration: ChiCTR2500104163). In addition to patients with AMI, we also included a comparative subgroup of patients with stable and unstable angina.

From 29 April 2025 to 31 August 2025, 158 participants were enrolled: 63 healthy controls, 15 patients with UA, and 80 consecutive AMI patients who underwent PCI within 12 h of symptom onset. Patients were enrolled based on the following diagnostic criteria: (1) sustained chest pain lasting over 30 min; (2) ST-segment elevation in more than two contiguous ECG leads; (3) angiographic confirmation of severe stenosis (>90%) in the culprit vessel; and (4) serum cardiac troponin T (cTnT) levels increased to twice the upper reference limit. Healthy controls were either verified free of cardiovascular disease or had <50% stenosis in all coronary segments at elective angiography. UA patients were required to have new-onset or accelerating angina within the preceding month, occurring at rest or with minimal exertion, without an associated cTnT elevation. Exclusion criteria were (1) prior myocardial infarction or stroke; (2) systemic autoimmune or inflammatory disorders; (3) cardiomyopathy, myocarditis or pericarditis; (4) severe liver or renal dysfunction; and (5) inability to provide written informed consent.

Baseline demographic information, laboratory results, and imaging findings were recorded for every participant. In-hospital adverse cardiovascular events were recorded, and participants were further followed at 30 days post-discharge. Adverse cardiovascular events were defined as: (1) malignant arrhythmia; (2) incident acute heart failure; (3) new or worsening left-ventricular systolic dysfunction (LVEF < 50% or ≥10% absolute decline from baseline); (4) recurrent myocardial infarction; and (5) all-cause mortality.

Peripheral blood was drawn as early as feasible after coronary intervention (within 12 h). To characterize the time-dependent release of tsRNA-3025a, a subset of AMI patients underwent serial sampling: on admission (immediately before PCI), and at 24 h, 48 h and 72 h after the procedure.

This study was conducted in accordance with the Declaration of Helsinki (1975 revision) and received approval from the Ethics Committee of Tongren Hospital, Shanghai Jiaotong University School of Medicine (approval No.: K2025-032-01) and Wuxi No. 2 People’s Hospital (approval No.: 2025 Y-3).

### 2.2. tsRNA Sequencing and Expression Analysis

Total RNA was isolated from the left ventricular tissues distal to the ligation site of sham and I/R-operated male mice (*n* = 3 per group). Subsequently, the quantity and integrity of the RNA samples were verified using a Qubit 3.0 Fluorometer (Thermo Fisher Scientific, Waltham, MA, USA). Given that certain RNA modifications can interfere with adaptor ligation, we utilized the RNA Pretreatment Assay (Arraystar, Rockville, MD, USA) to eliminate 3′-aminoacyl, 3′-cyclic phosphate, 5′-hydroxyl, m1A, and m3C modifications prior to library construction. Following the successive ligation of 3′ and 5′ adaptors, the constructs were reverse transcribed to generate cDNA, which was subsequently enriched via PCR using RT primers compatible with the Illumina platform. Target fragments ranging from 135 to 160 bp were isolated via polyacrylamide gel electrophoresis. The Illumina NextSeq 500 platform (Illumina, San Diego, CA, USA) was utilized to execute the sequencing runs. Following the initial quality assessment using FastQC software (version 0.12.1), the raw sequencing reads underwent trimming to remove adaptor sequences and low-quality bases via Cutadapt (version 4.7). The cleaned reads were subsequently mapped to the mouse reference genome (mm10) and genomic references encompassing both mature and precursor tRNAs obtained from the GtRNAdb, tRFdb, and MINTbase databases using the BWA algorithm (version 0.7.17). Finally, the aligned reads were utilized to quantify tRF/tiRNA abundance. Differential expression analysis between the I/R and sham groups was performed using the DESeq2 package in R (version 4.3.2). Differentially expressed tsRNAs were identified based on the thresholds of a fold change >2.0 and a false discovery rate (FDR) < 0.05.

Validation of the tsRNA-3025a expression profile was conducted in an independent cohort of mice (*n* = 6 per group) to confirm the initial sequencing findings.

### 2.3. Plasma RNA Extraction

Plasma samples (250 µL) were homogenized with 750 µL of TRIzol LS (Thermo Fisher Scientific) and incubated at room temperature for 5 min. To normalize variation, 4 µL of synthetic cel-miR-39-3p (200 nM) was spiked into the lysate as an exogenous control. Phase separation was achieved by chloroform addition, vigorous shaking (15 s), and centrifugation (12,000× *g*, 15 min), after which the supernatant was transferred. To enhance RNA recovery, precipitation was carried out using 4 µL of the ethachinmate carrier (Takara, Otsu, Japan) and 1/10 of the volume of 3 M sodium acetate, followed by the standard manufacturer’s purification steps.

### 2.4. Reverse Transcription and RT-qPCR Analysis

To quantify tsRNA expression, cDNA was constructed utilizing the stem-loop or tailing A miRNA First-Strand cDNA Synthesis Kit (#MR101/#MR201, Vazyme, Nanjing, China). Subsequent qPCR assays were conducted with the Unimodal SYBR qPCR Master Mix (#MQ102, Vazyme). The comparative cycle threshold (ΔCt) method was employed to determine relative abundance, with endogenous U6 serving as the normalizer. To quantify mRNA expression, cDNA synthesis and subsequent qPCR were conducted utilizing HiScript III All-in-one SuperMix (#R333, Vazyme) and ChamQ Universal SYBR qPCR Master Mix (#Q711, Vazyme), with β-Actin serving as the endogenous reference. All real-time PCR assays were executed on the QuantStudio 3 System (Applied Biosystems, Waltham, MA, USA). Detailed information regarding the primer sequences is available in Supplementary Table S4.

### 2.5. Cell Culture and Oxygen-Glucose Deprivation/Reperfusion (OGD/R) Treatment

AC16 cells were purchased from Shanghai Zhongqiaoxinzhou Biotech (Shanghai, China) and maintained in DMEM/F-12 medium (#SH30023.01, HyClone, Logan, UT, USA) containing 10% fetal bovine serum (FBS; #10099141C, Gibco, Grand Island, NY, USA) at 37 °C with 5% CO_2_. To simulate I/R injury in vitro, hypoxic conditions were generated using a trigas incubator (Esco, CCL-050 T-8, Singapore). Cells were washed and switched to glucose- and serum-free medium, then exposed to hypoxia (0.1% O_2_, 5% CO_2_, 95% N_2_) for 6 h. Notably, the induction medium was pre-equilibrated in the hypoxic chamber for 24 h to ensure oxygen depletion. Reperfusion was subsequently initiated by replacing the medium with fresh complete medium and incubating cells under normoxic conditions at 37 °C for 4 h.

### 2.6. RNA Synthesis and Transfection

Synthesized tsRNA-3025a mimic and inhibitor (featuring 2′-O-Me modification), as well as agomir and antagomir (conjugated with 3′Cholesteryl), were obtained from Sangon Biotech (Shanghai, China). For in vitro studies, oligonucleotide transfection was facilitated by Lipofectamine RNAiMAX (#13778075, Thermo Fisher Scientific). Detailed sequence information for all constructs is provided in Supplementary Table S6.

### 2.7. Assessment of Proliferation, Apoptosis, and Viability

To assess cell growth, metabolic activity was monitored using the Cell Counting Kit-8 (CCK-8; #HY-K0301, MedChemExpress, Monmouth Junction, NJ, USA), where the 450 nm optical density was captured by a BioTek FLx8 microplate reader (Winooski, VT, USA). Apoptotic rates were determined after labeling cells with an Annexin V-FITC/PI detection kit (#PF00005, Proteintech, Rosemont, IL, USA), followed by data acquisition on a BD FACSVerse™ flow cytometry system (Franklin Lakes, NI, USA). Furthermore, cytotoxicity and cellular viability were quantified via an LDH Release Assay and AO/PI staining (Beyotime, Shanghai, China), respectively, in accordance with the producer’s guidelines.

### 2.8. Mitochondrial Membrane Potential

Mitochondrial membrane potential (ΔΨm) was evaluated with the JC-1 Assay Kit (#HY-15534, MedChemExpress). After OGD/R modeling, AC16 cells were stained with JC-1 and imaged by a confocal microscope under identical acquisition settings, and fluorescence intensity was quantified in ImageJ (version 2.16.0) (NIH) to assess ΔΨm.

### 2.9. RNA Fluorescence In Situ Hybridization (FISH)

To visualize the localization of tsRNA-3025a, RNA-FISH was executed using a commercial kit (Ribobio, Guangzhou, China) following the provided instructions. Custom-designed Digoxigenin-labeled probes, specifically targeting the junction sequence of tsRNA-3025a, were synthesized by Sangon Biotech (Shanghai, China). AC16 cells were initially seeded in 24-well plates containing coverslips at a density of 3 × 10^4^ cells per well. Once the cell density reached 60–70% confluence, fixation was performed with 4% paraformaldehyde for 10 min at room temperature. Subsequently, cells were rinsed with PBS and permeabilized for 5 min at 4 °C using 0.5% Triton X-1005. After a 30 min blocking step at 37 °C in pre-hybridization buffer, the samples underwent overnight incubation at 37 °C in a dark environment with the hybridization solution (comprising 2.5 μL of probe mix in 100 μL of buffer). Following thorough washing in the dark, nuclei were visualized with DAPI staining (Beyotime), and the coverslips were mounted using antifade medium (Beyotime) before fluorescence microscopy imaging.

### 2.10. Isolation of the Nuclear and Cytoplasmic Fractions

Cytoplasmic and nuclear fractions of AC16 cells were separated utilizing the Nuclear and Cytoplasmic Extraction Kit (#P0027, Beyotime). To prevent RNA degradation during lysis, the extraction reagents were supplemented with RNase Inhibitor (#R0105, Beyotime) to a final concentration of 2 U/µL and DNase-RNase free DL-Dithiothreitol (DTT) to 0.8 mM. Following fractionation, the relative abundance of tsRNA-3025a, U6 (nuclear marker), and GAPDH (cytoplasmic marker) was determined via RT-qPCR.

### 2.11. Animal Experiments

Study protocols adhered strictly to the NIH Guide for the Care and Use of Laboratory Animals. To establish the myocardial I/R model, mice were subjected to anesthesia using 2% isoflurane. Following a left-sided intercostal incision to reveal the thoracic cavity, the heart was surgically exposed. We then initiated ischemia by snaring the left anterior descending (LAD) coronary artery with a 7-0 nylon suture (Jinhuan Medical, Shanghai, China) formed into a slipknot. Prior to ligation, tsRNA-3025a expression was modulated by intramyocardial injection of agomirs or antagomirs (40 nmol in 15 μL of PBS) alongside their respective negative controls. Reperfusion was initiated by releasing the knot after 45 min of ischemia. Then, mice were randomly assigned to different experimental cohorts after the surgical procedures. While these animals underwent surgery in the same batch to minimize technical variation, distinct cohorts were utilized for terminal (e.g., TTC staining, IF, and TEM) and longitudinal (Masson staining) assessments to ensure that data were obtained from independent biological replicates.

At 24 h post-reperfusion, mice were euthanized (5% isoflurane). Serum was isolated by centrifugation (2000× *g*, 10 min) to quantify LDH and creatine kinase-MB (CK-MB) levels using a Sysmex BX-3010 analyzer (Kobe, Japan). Myocardial infarct size was identified through Evans blue perfusion (#HY-B1102, MedChemExpress) and subsequent staining with 2,3,5-triphenyltetrazolium chloride (TTC, #HY-D0714, MedChemExpress). For histological examination, the heart specimens were embedded in paraffin blocks and sliced at a 5 μm thickness. H&E staining was utilized to observe pathological alterations, whereas fibrotic changes in the heart were analyzed 28 days after I/R via Masson’s trichrome staining. Cellular apoptosis was quantified using a commercial TUNEL Assay Kit (#C1089, Beyotime), adhering to the procedures specified by the manufacturer.

### 2.12. Echocardiography

Cardiac function was assessed 24 h following I/R using a Vevo-2100 imaging system (FUJIFILM VisualSonics Inc., Toronto, ON, Canada). Throughout the examination, mice were anesthetized with 1.5% isoflurane and allowed to breathe spontaneously, ensuring heart rates stayed within a physiological range of 400–550 bpm. M-mode recordings were obtained at the level of the papillary muscles via a two-dimensional short-axis orientation. Data were averaged over five consecutive cardiac cycles to calculate left ventricular ejection fraction (LVEF), left ventricular fractional shortening (LVFS), and left ventricular end-diastolic diameter (LVEDD).

### 2.13. Transmission Electron Microscopy (TEM)

Heart tissues were harvested from border zone between infarcted and non-infarcted regions. The specimens were washed with 0.1 M phosphate buffer (pH 7.2) and immediately submerged in ice-cold 2.5% glutaraldehyde (PBS, pH 7.0) for fixation at 4 °C for 12 h. Post-fixation was performed using 1% osmium tetroxide for 1 h. Subsequent to the dehydration and resin embedding phases, ultrathin slices (70–90 nm) were sectioned and subjected to counterstaining with uranyl acetate and lead citrate. An HT7800 transmission electron microscope (Hitachi, Tokyo, Japan) was employed to record the ultrastructural images. Mitochondrial abnormalities, such as cristae loss and swelling, were evaluated in three randomly chosen fields per section.

### 2.14. Luciferase Assay

To verify direct binding between tsRNA-3025a and the PIK3C2A 3′untranslated region (UTR), dual-luciferase assays were performed. The PIK3C2A 3′UTR sequence was inserted into the PGL3-CMV-LUC-MCS vector. Cells were co-transfected with the reporter plasmids (wild-type or mutant), pRL-TK (internal control), and either tsRNA-3025a mimics or negative control (NC) mimics. Forty-eight hours post-transfection, cells were lysed, and luciferase activity was quantified using a Promega kit (Madison, WI, USA) per the manufacturer’s manual. Luminescence was recorded on a FLUOstar Omega reader (BMGLABTECH, Offenburg, Germany), with Firefly luciferase activity normalized to Renilla signals.

### 2.15. Western Blotting

To isolate total protein, cells were subjected to lysis in RIPA buffer (#P0013C, Beyotime) supplemented with a protease inhibitor cocktail (#P1008, Beyotime) for 30 min on ice. After centrifuging the lysate at 13,000× *g* for 20 min, we determined the protein levels in the resulting supernatant using a BCA Protein Assay Kit (#ZJ101, Epizyme, Shanghai, China). Protein samples (20 μg per lane) were separated via SDS-PAGE and subsequently electroblotted onto PVDF membranes (Millipore, Billerica, MA, USA). Following a blocking step with 5% skimmed milk, the membranes were incubated with specific primary antibodies at 4 °C overnight. Loading controls included β-Tubulin (#ab6046, Abcam, Cambridge, United Kingdom) and β-actin (#20536-1, Proteintech). Other targets were detected using antibodies against Caspase-3 (#19677-1-AP, Proteintech), Bax (#50599-2, Proteintech), PI3K-c2α (#22028-1-AP, Proteintech), LC3B (#ET1701-65, HUABIO, Hangzhou, China), and p62 (#HA721171, HUABIO).

### 2.16. Autophagic Flux Assay

To monitor autophagic flux, AC16 cells were infected with Lentivirus-Stub-RFP-Sens-GFP-LC3 (Genechem, Shanghai, China) at a multiplicity of infection (MOI) of 20. After a 12 h incubation, the medium was refreshed, and cells were cultured for another 48 h. Stable cell lines were subsequently established via selection with puromycin (#ST551, Beyotime Biotechnology). In this reporter system, the accumulation of red LC3 puncta signifies the formation of acidic autolysosomes (intact flux), whereas green fluorescence indicates the retention of autophagosomes, reflecting impaired lysosomal fusion or flux blockage.

### 2.17. RNA Sequencing and Bioinformatic Analysis

AC16 cells were pretreated with tsRNA-3025a inhibitors or negative control (NC) inhibitors for 24 h prior to OGD/R exposure. Total RNA was isolated using TRIzol from three groups: Control, OGD/R+NC inhibitor, and OGD/R+tsRNA-3025a inhibitor. Library construction and high-throughput sequencing were outsourced to OE Biotech Co., Ltd. (Shanghai, China). For data analysis, differentially expressed genes (DEGs) were screened using the DESeq2 package with cutoffs of |log2FC| > 1.0 and *p* < 0.05. Downstream functional annotation, including Gene Set Enrichment Analysis (GSEA) and KEGG pathway mapping, was performed in R (version 4.3.2) via hypergeometric testing.

### 2.18. Sample Size Calculation

The sample size was calculated using PASS 2023 software (NCSS, LLC, Kaysville, UT, USA). The calculation was based on the Two-Sample *t*-tests Allowing Unequal Variance method. Parameters were set with a two-sided significance level (α) of 0.05 and a power (1 − β) of 0.90. Based on preliminary pilot data, the mean and standard deviation of plasma tsRNA-3025a values (−ΔCT) were 0.0596 ± 2.11577 for the healthy control group and 4.7637 ± 2.39970 for the AMI group. Assuming a 1:1 allocation ratio, the initial calculation indicated a requirement of 7 participants per group. To account for a potential 20% dropout rate, the minimum total sample size was established at 18 participants.

### 2.19. Statistical Analysis

Patient characteristics are summarized as medians with interquartile ranges (IQRs) for continuous variables, while categorical data are reported as frequencies and percentages. Other experimental results are expressed as the mean ± standard error of the mean (SEM). For continuous data, differences between two groups were evaluated using Student’s *t*-test, whereas one-way ANOVA with Tukey’s post hoc test was applied for multiple-group comparisons. Categorical variables were compared utilizing either Pearson’s chi-squared test or Fisher’s exact test. To determine independent predictors for elevated tsRNA-3025a and major adverse cardiovascular events (MACEs), logistic regression models were constructed. Variables yielding a *p*-value < 0.1 in the univariate analysis were subsequently included in the multivariate regression model. All analyses were conducted using SPSS software (version 27.0; SPSS Inc., Chicago, IL, USA), with statistical significance defined as *p* < 0.05. Experiments were performed in triplicate at minimum.

## 3. Results

### 3.1. I/R Induces Cardiac Injury with Alternations in tsRNA Profile in Mice

First, C57BL/6 mice were subjected to an I/R model. Tissue and blood were collected on days 1, 2 and 3 post-operations. Then, echocardiographic analysis revealed that LVEF, LVFS, and LVEDD were significantly decreased in the I/R group (Figure S1A). Next, hearts were harvested for histopathological evaluation, with H&E staining revealing disrupted myocardial architecture, and TUNEL staining showing extensive cardiomyocyte apoptosis in the I/R region, collectively confirming successful establishment of the model (Figure S1B,C).

**Figure 1:**
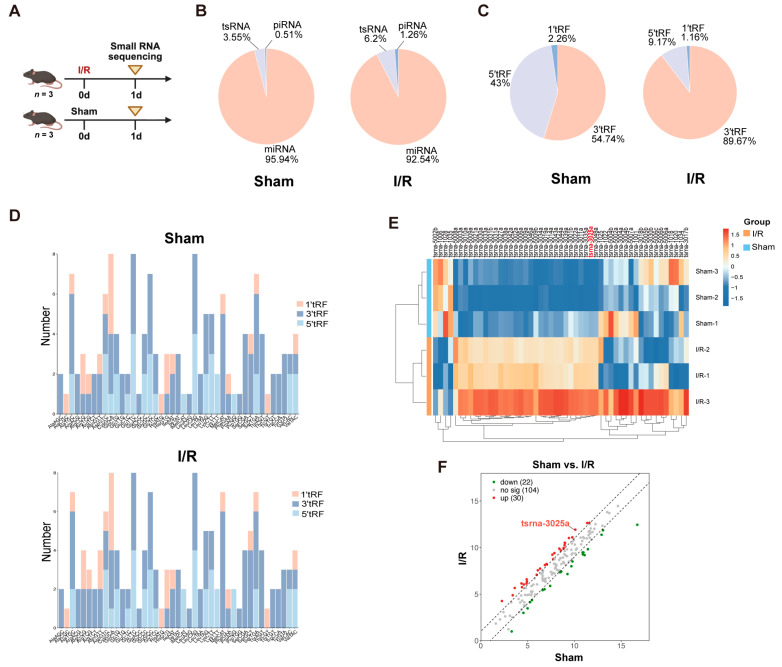
I/R induces cardiac injury with alternations in tsRNA profile in mice. (**A**) Schematic diagram of the experimental timeline. Mouse hearts were harvested at 1 day post-I/R surgery for small RNA sequencing analysis. (**B**) Distribution of three major small non-coding RNA classes in sham (*n* = 3) and I/R (*n* = 3) groups. (**C**) Relative proportions of three primary tsRNA subtypes in sham and I/R groups. (**D**) Subtype-specific tsRNA profiles derived from individual tRNAs in sham and I/R groups. (**E**) Cluster heatmap of significantly regulated tsRNAs in I/R mice subjected to 45 min of ischemia and 24 h of reperfusion. (**F**) Scatter plot of differential tsRNA expression. Red dots indicate upregulated tsRNAs, while green dots denote downregulated tsRNAs in sham and I/R groups. The dashed lines parallel to the diagonal indicate the fold-change thresholds (FC > 2.0 or FC < 0.5). Data are expressed as mean ± SE. I/R, ischemia–reperfusion; tsRNA, tRNA-derived small RNA.

Furthermore, small RNA sequencing was performed to compare cardiac small RNA profiles between sham and I/R groups. The relative abundance of miRNAs and tsRNAs was markedly increased in I/R hearts compared to the sham group, suggesting that these non-coding RNAs may contribute to the development of I/R injury (Figure 1B). A substantial difference in tsRNA expression was observed between groups: 52 tsRNAs were differentially expressed (|log2FC| ≥ 1), with 30 upregulated and 22 downregulated in I/R versus sham. These changes are visualized in a hierarchical clustering heatmap (Figure 1E). Subtype analysis of tsRNA revealed that the proportion of 3′-tRFs increased by 34.94% in I/R compared to sham (Figure 1C). Among source tRNAs, tsRNAs derived from tRNA-Ser were most abundant (Figure 1D). We further focused on the top three differentially expressed tsRNAs, along with tsRNA-3025a, a 3′-tRF derived from tRNA-Ser, which was markedly upregulated in I/R hearts, as shown in the scatter plot (Figure 1F). A previous study reported that 3′-tRFs regulate mRNA expression through RNA–protein interactions, especially in cancer and epigenetic contexts [25], supporting the biological relevance of tsRNA-3025a for further functional investigation. To validate the sequencing results, we quantified the three most upregulated tsRNAs, together with tsRNA-3025a, in independent murine and human blood samples.

### 3.2. tsRNA-3025a Upregulates in Ischemia–Reperfusion Injury

**Figure 2:**
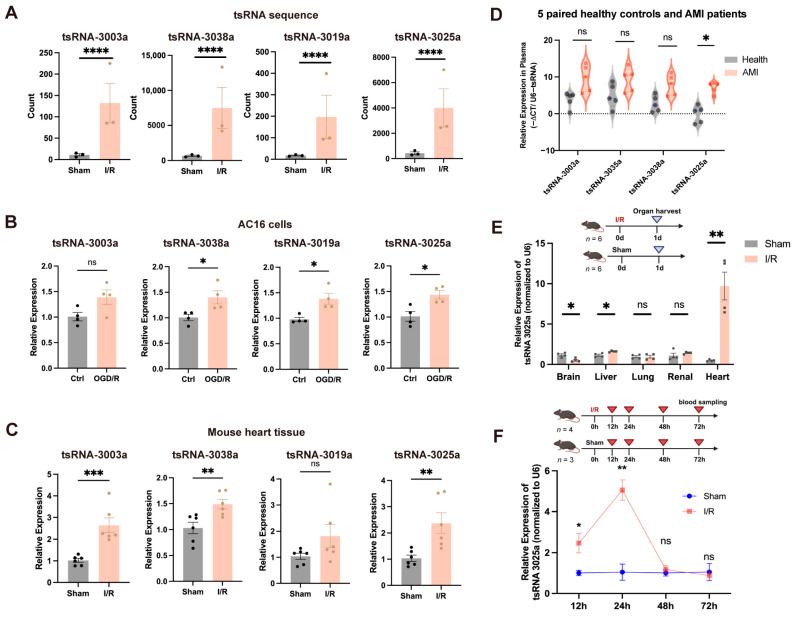
tsRNA-3025a upregulates in ischemia–reperfusion injury. (**A**) The small RNA count results from tsRNA-seq. (**B**–**D**) The expression of the top 4 selected tsRNA was validated using qRT-PCR in OGD/R-treated AC16 cells (**B**), heart tissue of the I/R group compared with the sham group (*n* = 6 per group) (**C**), and 5 pairs of plasma samples from AMI patients and healthy controls (**D**). (**E**) The expression levels of tsRNA-3025a in various organs at 1 day post-I/R detection by qRT-PCR. (**F**) Relative expression levels of tsRNA-3025a in mouse plasma at 12, 24, 48, and 72 h post-I/R. Blood samples were collected via the retro-orbital sinus at the indicated time points. Data from the I/R group (*n* = 4) are compared to the time-matched sham group (*n* = 3). All data are representative of at least three independent experiments. Data are expressed as mean ± SE. * *p* < 0.05, ** *p* < 0.01, *** *p* < 0.001, **** *p* < 0.0001, ns indicates not significant. Results of tsRNA levels are normalized to U6. I/R, ischemia–reperfusion; OGD/R, oxygen-glucose deprivation/reperfusion; tsRNA, tRNA-derived small RNA; AMI, acute myocardial infarction.

To evaluate whether tsRNA expression under pathological conditions is consistent with the sequencing results, we detected the top three upregulated tsRNAs (tsRNA-3003a, tsRNA-3038a, tsRNA-3019a) identified by tsRNA-seq, as well as tsRNA-3025a, across the murine myocardial I/R model, the oxygen–glucose deprivation/reoxygenation (OGD/R)-treated AC16 cell model, and plasma from patients with AMI. In the tsRNA-seq dataset, read counts for tsRNA-3003a, tsRNA-3038a, and tsRNA-3019a were significantly higher in I/R hearts (Figure 2A). In OGD/R-treated AC16 cells, tsRNA-3003a showed an upward trend but did not reach statistical significance (Figure 2B). Similarly, in an independent validation using mouse heart tissue, tsRNA-3019a exhibited comparable expression between groups (Figure 2C). We therefore measured the remaining candidates in paired serum samples from five AMI patients and matched healthy controls; only tsRNA-3025a was significantly elevated (Figure 2D). To explore temporal dynamics and tissue specificity, we detected tsRNA-3025a in multiple organs from I/R mice and in plasma collected at 12, 24, 48, and 72 h after reperfusion. Notably, tsRNA-3025a displayed cardiac specificity, with a marked increase confined to I/R mouse heart tissue (Figure 2E). In plasma, tsRNA-3025a rose by 12 h, peaked at 24 h, and returned to baseline by 48 h, suggesting a prompt response relative to traditional myocardial injury markers (Figure 2F).

### 3.3. Characteristic and Comparison of Detection Methods of tsRNA3025a

**Figure 3:**
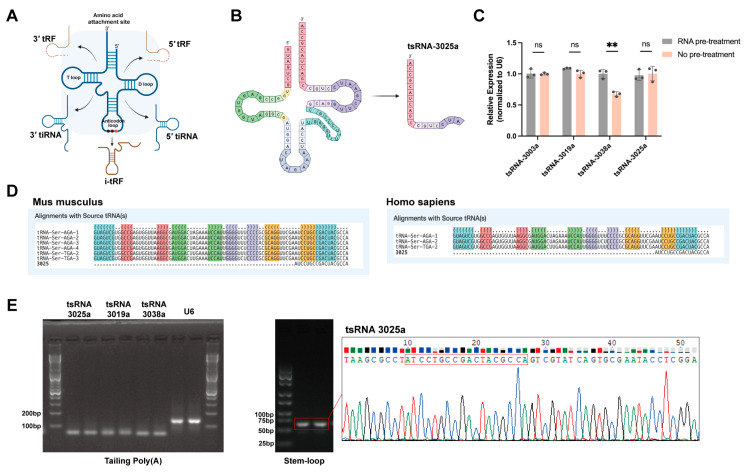
Characteristics and comparison of detection methods of tsRNA3025a. (**A**) tsRNAs comprise five main subtypes: 3′-tRF, 5′-tRF, i-tRF, 3′-tiRNA, and 5′-tiRNA. (**B**) tsRNA-3025a is identified as a 3′-tRF derived from tRNA-Ser with a length of 18 nt. (**C**) Relative expression levels of tsRNA with or without RNA-pretreatment kit using Stem loop qRT-PCR. (**D**) tsRNA-3025a is derived from tRNA-Ser-AGA and tRNA-Ser-TGA, which exhibit sequence homology and complete identity between mice and humans. Distinct structural regions are color-coded: cyan, acceptor stem; red, D-arm; green, anticodon arm; purple, variable arm; and yellow, T-arm. (**E**) PCR products generated by poly(A)-tailing qRT-PCR (left) and stem-loop qRT-PCR (right) are visualized by agarose gel electrophoresis. The red box indicates the stem-loop product of tsRNA-3025a, which was confirmed by Sanger sequencing. tRF, tRNA-derived fragment; tiRNA, tRNA-derived and stress-induced small RNA. Data are expressed as mean ± SE. ** *p* < 0.01, ns indicates not significant.

Based on specific cleavage sites, tsRNAs are generally categorized into two primary lineages: tiRNAs (5′- and 3′-tiRNAs) and tRFs (5′-tRFs, 3′-tRFs, and internal tRFs/i-tRFs) (Figure 3A). tsRNA-3025a, derived from mature tRNA-Ser-AGA/TGA, is an 18-nt 3′-tRF (Figure 3B). Notably, the tRNA-Ser-AGA/TGA sequences are identical in humans and mice, indicating evolutionary conservation and suggesting a potential role in reperfusion injury (Figure 3D).

tsRNAs carry extensive modifications that can impede cDNA synthesis, and many tsRNAs share identical core sequences; thus, traditional detection often involves demodification and 3′/5′ adaptor ligation. However, recent work shows that robust amplification can be achieved without demodification using stem-loop or poly(A)-tailing strategies [26]. Accordingly, we performed stem-loop RT–qPCR on the same tsRNA with and without an RNA pretreatment kit and observed comparable relative expression (Figure 3C). We performed reverse transcription of distinct tsRNAs using both stem-loop and poly(A) tailing approaches. Following PCR amplification, agarose gel electrophores were revealed as single discrete bands for all tsRNAs, including the U6 reference. This demonstrates the specificity of amplification products generated by our correspondingly designed primer pairs for each method (Figure 3E, left). Finally, Sanger sequencing of the stem-loop RT–qPCR production confirmed an exact sequence match (Figure 3E, right).

### 3.4. Elevated tsRNA-3025a Is Associated with Serious Cardiac Injury in ACS Patients and Predicts Adverse Events

**Figure 4:**
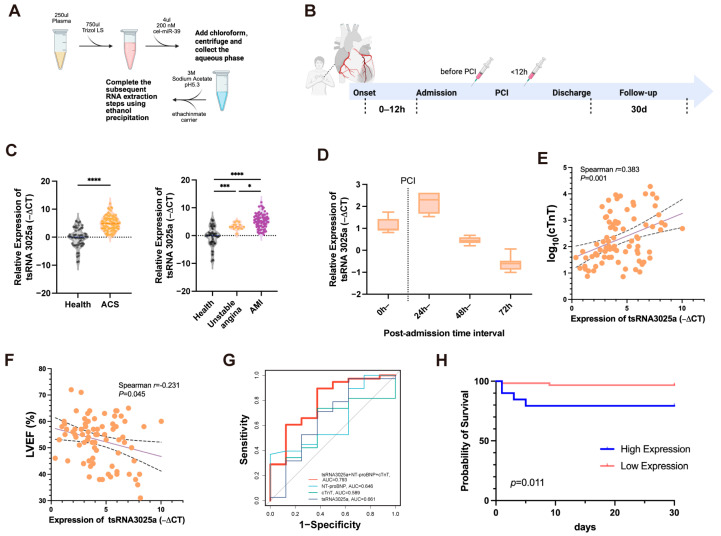
Elevated tsRNA-3025a is associated with serious cardiac injury in ACS patients and predicts adverse events. (**A**) Schematic of the workflow for extracting cell-free tsRNAs from human plasma. (**B**) Schematic of the clinical study design showing enrollment of patients with ACS and collection of blood samples and clinical data before and after reperfusion therapy. (**C**) Left: Relative expression of tsRNA-3025a in healthy controls (*n* = 63) versus ACS patients (*n* = 95). Right: Relative expression of tsRNA-3025a in healthy controls (*n* = 63), unstable angina (*n* = 15), and AMI (*n* = 80). (**D**) Temporal changes in the relative expression of tsRNA-3025a in AMI patients from pre-revascularization to 72 h post-revascularization. (**E**,**F**) Scatter plots showing Spearman correlations between tsRNA-3025a levels and cTnT (**E**) and LVEF (**F**). The solid line represents the linear regression fit, and the dashed lines indicate the 95% confidence interval. (**G**) ROC curves of tsRNA-3025a, NT-proBNP, and cTnT for predicting adverse cardiovascular events occurring during hospitalization and within 1 month after discharge. Events include acute heart failure exacerbation, LVEF < 50% or >10% decrease from baseline, malignant arrhythmia, reinfarction, and death. (**H**) Survival curve analysis of AMI patients stratified by high versus low tsRNA-3025a expression, with the cutoff defined as the 75th percentile of relative expression. Data are expressed as mean ± SE. * *p* < 0.05, *** *p* < 0.001, **** *p* < 0.0001, ns indicates not significant. Statistical significance uses one-way ANOVA with Tukey’s post hoc test. ACS, acute coronary syndrome; AMI, acute myocardial infarction; NT-proBNP, n-terminal b-type natriuretic peptide; cTnT, cardiac troponin T; ROC, receiver operating characteristic; LVEF, LV ejection fraction.

Given the rapid elevation of tsRNA-3025a in plasma with promising clinical implications, we initiated a prospective cohort study to evaluate its clinical value. Since circulating RNA levels in fresh plasma are lower than those in tissues, we optimized the methodology for efficient RNA extraction and added exogenous controls (cel-miR-39-3p) for quality assurance (Figure 4A). Figure 4B outlines the design of the observational clinical study cohort.

#### 3.4.1. Clinical Characterization and Temporal Expression Profiles of Circulating tsRNA-3025a in ACS Patients

Figure S2 describes the entire enrollment process. In brief, two participants were excluded due to suspected CAD in the control group. In the AMI group, three participants did not complete blood sample collection, and two were excluded due to failure to complete follow-up. In the UA group, five participants were excluded due to elevated troponin T. Table 1 and Table S1 provide a detailed overview of the baseline profiles and clinical data for healthy controls, AMI patients, and UA patients. AMI patients had significantly higher rates of coronary risk factors (smoking, hyperlipidemia, diabetes, and hypertension) compared to healthy controls. The median time from symptom onset to hospital admission was within 6 h, and patients with outdated myocardial infarction were not included in the study. Overall, tsRNA-3025a expression was significantly higher in ACS patients compared to healthy controls (Figure 4C, left). Among them, AMI patients exhibited the most significant and pronounced increase in tsRNA-3025a levels (Table 1). Although UA patients did not show overt coronary occlusion or myocardial infarction, their plasma tsRNA-3025a levels were mildly elevated compared to stable angina controls, with no changes in troponin and CK-MB (Table S1; Figure 4C, right). To explore the temporal expression of tsRNA-3025a, we tracked 7 AMI patients and collected blood samples at pre-PCI and at several time points post-PCI. Results showed that tsRNA-3025a peaked within 24 h of reperfusion and returned to baseline by 48 h, resembling the rapid rise and fall observed in animal models (Figure 4D).

#### 3.4.2. Association Between tsRNA-3025a and Cardiac Injury in Humans

Spearman correlation analysis indicated a positive correlation between tsRNA-3025a and myocardial injury markers, including troponin (Spearman r = 0.383) (Figure 4E). Furthermore, tsRNA-3025a showed a negative correlation with LVEF (Spearman r = −0.231) (Figure 4F). In addition, we classified patients into high-tsRNA-3025a-expression (those with −ΔCT above the 75th percentile) and low-expression (below the 75th percentile) groups. The high-expression group was younger, had a higher incidence of hyperlipidemia, and showed lower LVEF. Additionally, these patients exhibited worse laboratory markers, including elevated cTnT and LDL-C levels (Table 2). After adjusting for common clinical confounders (such as body weight and reperfusion time), multivariate logistic regression analysis identified independent risk factors for elevated tsRNA-3025a, including younger age (OR 0.945, 95% CI 0.896–0.998), higher preoperative Killip class (OR 2.058, 95% CI 1.076–3.936), triglyceridemia (2.682, OR 1.436–5.012), and lower ejection fraction (OR 0.890, 95% CI 0.805–0.984) (Table S2). We used N-terminal pro-brain natriuretic peptide (NT-proBNP) (AUC 0.646), cTnT (AUC 0.589), and tsRNA-3025a (AUC 0.661) to predict the risk of post-AMI heart failure and found that combining these three markers improved the AUC to 0.793 (Figure 4G).

#### 3.4.3. Association Between tsRNA-3025a and Adverse Events

The cohort study followed participants from hospital admission to 30-day post-discharge follow-up. During this period, seven AMI patients experienced adverse events: three developed severe malignant arrhythmias (one subsequently developed acute left heart failure), and four developed acute heart failure or worsening of heart function (one of whom later died). Adverse events were predominantly observed 1–3 days post-revascularization. Kaplan–Meier survival analysis revealed that patients with high tsRNA-3025a expression had a significantly higher rate of adverse events than those with low expression (Figure 4H). Multivariate logistic regression showed independent risk factors for MACEs, including younger age (OR 1.115, 95% CI 1.001–1.243), worse cardiac function (OR 0.885, 95% CI 0.797–0.983) and higher tsRNA-3025a (2.174, OR 1.238–3.816) (Table S3). Therefore, tsRNA-3025a shows great promise as a biomarker for risk stratification in AMI. It is closely associated with myocardial injury and adverse cardiovascular events.

### 3.5. tsRNA-3025a Promotes Apoptosis and Mitochondrial Membrane Depolarization In Vitro

**Figure 5:**
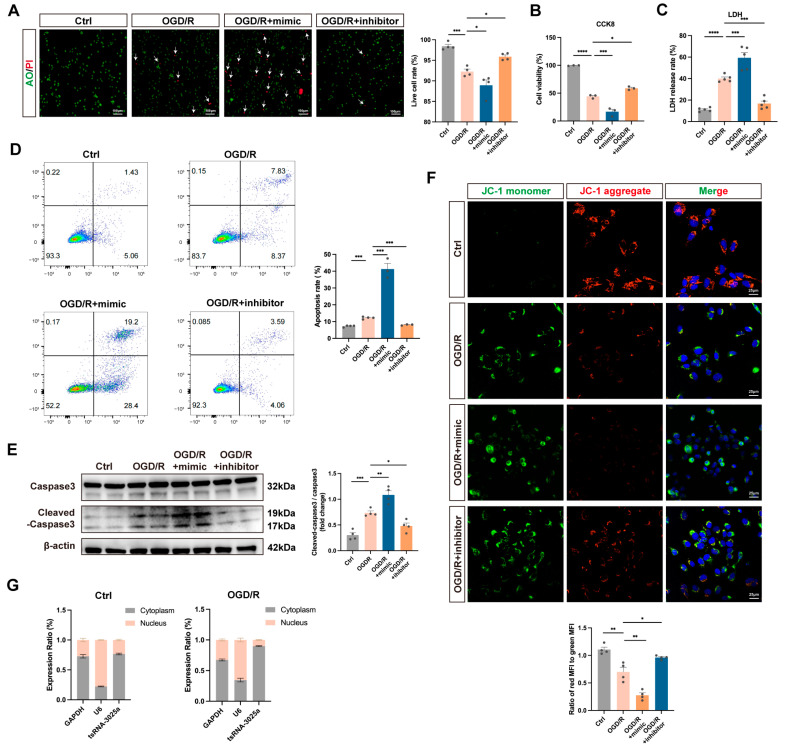
tsRNA-3025a promotes apoptosis and mitochondrial membrane depolarization in vitro. (**A**) Representative AO/PI staining images: red indicates dead cells (white arrow) and green indicates live cells. Scale bar: 100 µm. (**B**,**C**) CCK-8 cell viability (**B**) of OGD/R-treated AC16 cells with tsRNA-3025a overexpression or knockdown compared with control. LDH release ratio (**C**) in culture supernatants under the same conditions. (**D**) Flow-cytometric assessment of apoptosis by Annexin/PI staining after 6 h of OGD followed by 6 h of re-oxygenation in AC16 cells pretreated for 12 h to overexpress or knockdown tsRNA-3025a. (**E**) Western blot analysis of apoptosis markers Caspase-3 and cleaved Caspase-3, in control, OGD/R, OGD/R+inhibitor, and OGD/R+mimic groups. (**F**) JC-1 staining to evaluate mitochondrial membrane potential (ΔΨm) in control, OGD/R, and OGD/R with tsRNA-3025a overexpression or knockdown (scale bar = 25 µm). Red fluorescence indicates JC-1 aggregates (high ΔΨm), while green fluorescence indicates JC-1 monomers (low ΔΨm). (**G**) Nuclear-cytoplasmic fractionation showing relative distribution of tsRNA-3025a between nuclear and cytoplasmic compartments in control and OGD/R conditions. Data are expressed as mean ± SE. * *p* < 0.05, ** *p* < 0.01, *** *p* < 0.001, **** *p* < 0.0001. Statistical significance uses one-way ANOVA with Tukey’s post hoc test. All data are representative of at least three independent experiments. OGD/R, oxygen-glucose deprivation/reperfusion.

To clarify the mechanistic role of tsRNA-3025a, we conducted reperfusion injury in vitro using the OGD/R treatment in AC16 cells. A period of 24 h before OGD/R, cells were transfected with tsRNA-3025a mimic or inhibitor to achieve overexpression or knockdown, respectively. OGD/R significantly increased PI-positive cells, and this effect was alleviated by tsRNA-3025a knockdown (Figure 5A). Consistently, flow-cytometric analysis of Annexin V/PI-stained cells indicated that tsRNA-3025a overexpression increased both early and late apoptotic populations, whereas its silencing attenuated OGD/R-induced apoptosis (Figure 5D). In line with these findings, CCK-8 and LDH assays indicated that OGD/R decreased cell viability and increased extracellular LDH release; tsRNA-3025a overexpression further worsened these changes, while knockdown mitigated them (Figure 5B,C). At the protein level, canonical apoptosis markers, cleaved caspase-3 and Bax, were increased by tsRNA-3025a overexpression, and this effect was partially reversed by tsRNA-3025a knockdown (Figure 5E and Figure S3A). Fluorescence microscopy of JC-1-stained AC16 cells revealed that OGD/R caused a pronounced drop in mitochondrial membrane potential (ΔΨm). This loss was amplified by tsRNA-3025a overexpression but partially rescued by its knockdown after reperfusion (Figure 5F). FISH demonstrated that tsRNA-3025a was distributed predominantly within the cytoplasm, and the intensity of its cytoplasmic signal increased markedly after OGD/R (Figure S3B). To substantiate this localization, subcellular fractionation was performed to isolate RNA from nuclear and cytosolic lysates, and tsRNA-3025a abundance was quantified by real-time qPCR. Under normoxia conditions, tsRNA-3025a was enriched in the cytosolic portion, and the relative cytoplasmic level rose further following OGD/R injury (Figure 5G), consistent with the RNA FISH data.

### 3.6. tsRNA-3025a Aggravates I/R Injury in Mice, While Inhibiting tsRNA-3025a Alleviates Cardiac Injury

**Figure 6:**
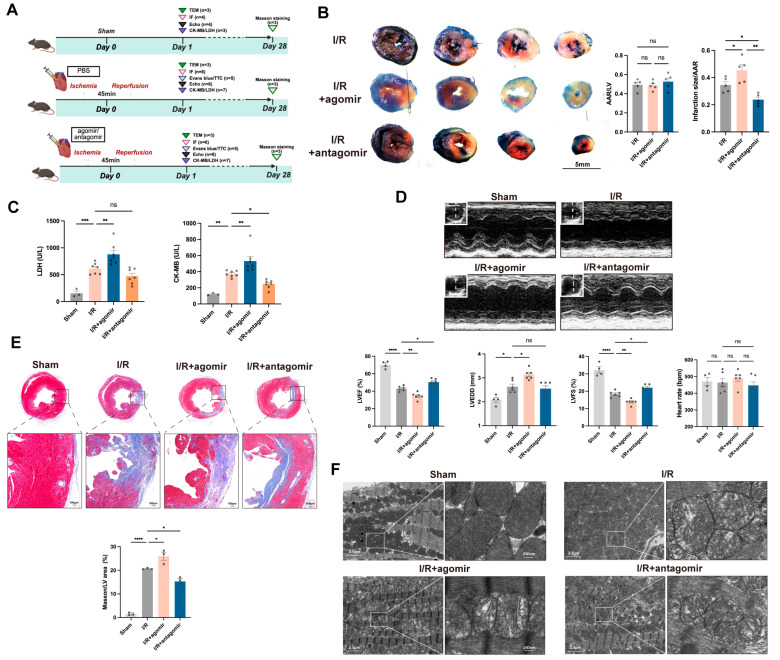
tsRNA-3025a aggravates I/R injury in mice, while inhibiting tsRNA-3025a alleviates cardiac injury. (**A**) Schematic representation of the in vivo experimental design. (**B**) Representative images and quantification of Evans blue/TTC-stained heart sections from mice subjected to myocardial I/R with tsRNA-3025a overexpression or knockdown (*n* = 5 per group). Blue regions represent the non-ischemic zone; the sum of red (viable myocardium) and white regions (infarcted myocardium) represents AAR. (**C**) Arterial blood collection with serum LDH and CK-MB measurement and quantification in the sham group (*n* = 3) and I/R with agomir or antagomir (*n* = 7 per group). (**D**) Representative echocardiography of sham (*n* = 4) and I/R mice with tsRNA-3025a knockdown or overexpression (*n* = 6 per group). EF, FS, and LVEDD were measured to evaluate the cardiac function. (**E**) Masson’s trichrome staining of cardiac sections 4 weeks after I/R (*n* = 3 per group, scale bar = 100 µm). Blue indicates collagen deposition, and red indicates viable myocardium. (**F**) Representative transmission electron microscopic images of mitochondria ultrastructure (*n* = 3 per group, scale bar = 2.5 µm, 250 nm). Data are expressed as mean ± SE. * *p* < 0.05, ** *p* < 0.01, *** *p* < 0.001, **** *p* < 0.0001, ns indicates not significant. Statistical significance uses one-way ANOVA with Tukey’s post hoc test. IF, immunofluorescence; I/R, ischemia–reperfusion; AAR, area at risk; LDH, lactate dehydrogenase; CK-MB, creatine kinase-MB; LVEF, LV ejection fraction; LVFS, LV fractional shortening; LVEDD, LV end-diastolic dimension.

To explore the function of tsRNA-3025a in vivo, we established a murine I/R model and performed intramyocardial injection at the intended ligation site immediately before LAD ligation to modulate tsRNA-3025a expression. tsRNA-3025a agomir or antagomir was injected to overexpress or knockdown tsRNA-3025a (Figure 6A). Evans blue/TTC dual staining showed that tsRNA-3025a overexpression increased infarct size, whereas knockdown mitigated reperfusion injury and increased the salvageable fraction (Figure 6B). Arterial blood was collected 24 h after reperfusion, and plasma LDH and CK-MB were measured. tsRNA-3025a knockdown reduced the level of LDH and CK-MB, and tsRNA-3025a overexpression further increased them (Figure 6C). At 24 h post-reperfusion, transthoracic echocardiography (heart rate maintained at 400–500 bpm) revealed depressed cardiac function in I/R mice; notably, the tsRNA-3025a antagomir improved LVEF and LVFS, whereas tsRNA-3025a overexpression further worsened cardiac function and increased LVEDD (Figure 6D). TUNEL staining demonstrated that tsRNA-3025a overexpression augmented cardiomyocyte apoptosis, while knockdown decreased the proportion of TUNEL-positive myocardium cells (Figure S4A). Masson’s trichrome staining at day 28 post-I/R showed increased fibrosis with tsRNA-3025a agomir and a protective reduction in fibrosis with tsRNA-3025a antagomir (Figure 6E). Immunofluorescence staining showed that the proportion of Ki67+ and pH3+ cardiomyocytes was higher in the tsRNA-3025a antagomir group than the I/R group (Figure S4B,C). Meanwhile, transmission electron microscopy revealed that tsRNA-3025a overexpression exacerbated I/R-induced ultrastructural damage, including disrupted myofibrillar integrity, mitochondrial swelling, cristae fragmentation, and vacuolization, whereas knockdown partially alleviated these abnormalities (Figure 6F). Collectively, these data indicate that under I/R stimulation, tsRNA-3025a aggravates myocardial injury, impairs cardiac function, and exacerbates mitochondrial structural damage. Knockdown of tsRNA-3025a promotes myocardial repair and proliferation following reperfusion injury.

### 3.7. tsRNA-3025a Is Involved in the Autophagy Pathway by Regulating the Expression of PIK3C2A

**Figure 7:**
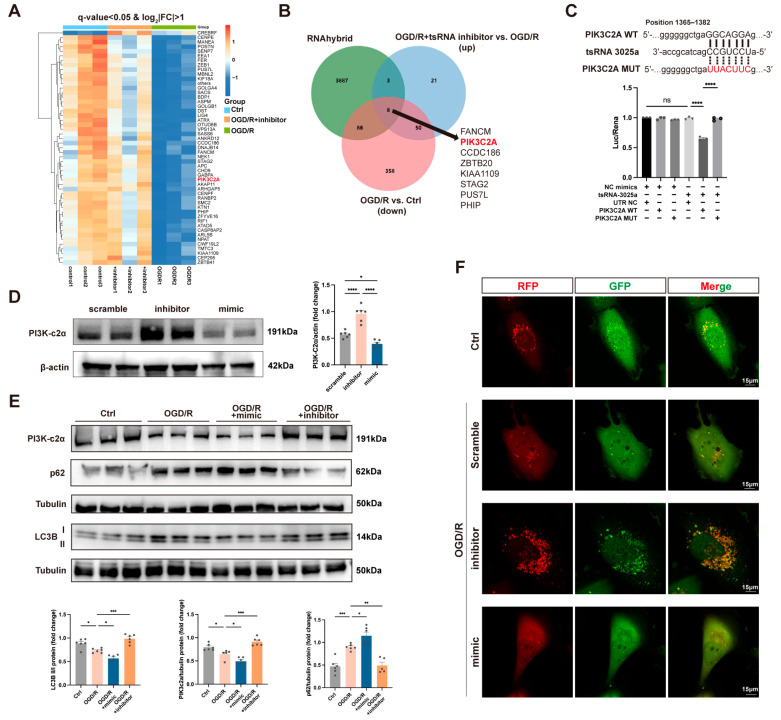
tsRNA-3025a is involved in the autophagy pathway by regulating the expression of PIK3C2A. (**A**) Hierarchical clustering heatmap from RNA-seq analysis comparing the OGD/R group with the OGD/R+tsRNA-3025a knockdown group. (**B**) The genes targeted by tsRNA-3025a were identified using RNAhybrid and transcriptome sequence analyses (downregulation in OGD/R vs. control, while upregulation in tsRNA knockdown treatment vs. OGD/R). Arrow indicated the 8 overlapping target genes. (**C**) Schematic of the PIK3C2A 3′UTR wild-type (wt) and mutant (mut) reporter constructs; the luciferase activity of pmirGLO-PIK3C2A is decreased by the tsRNA-3025a mimic in AC16 cells. (**D**) tsRNA-3025a overexpression or knockdown in AC16 cells decrease or increase PIK3C2A expression at the protein level. (**E**) Western blot analysis of PI3K-c2α, p62, and LC3B in AC16 cells transfected with tsRNA-3025a mimic or inhibitor. (**F**) Representative immunofluorescence images of AC16 cells transduced with a tandem fluorescent LC3B lentiviral reporter and subjected to OGD/R after tsRNA-3025a overexpression or knockdown (scale bar = 15 µm). Yellow puncta (GFP^+^/RFP^+^) indicate autophagosomes, and red puncta (GFP^−^/RFP^+^) represent autolysosomes. Data are expressed as mean ± SE. * *p* < 0.05, ** *p* < 0.01, *** *p* < 0.001, **** *p* < 0.0001, ns indicates not significant. Statistical significance uses one-way ANOVA with Tukey’s post hoc test. OGD/R, oxygen-glucose deprivation/reperfusion; GFP, green fluorescent protein; RFP, red fluorescent protein.

A heatmap displays the top 50 genes among the control, OGD/R and tsRNA-3025a inhibitor-treated OGD/R groups (Figure 7A). To find the principal downstream effector of tsRNA-3025a, we performed RNA-seq on AC16 cells from three conditions: control, OGD/R, and OGD/R+tsRNA-3025a inhibitor. A total of 1128 genes were differentially expressed (|log2FC| ≥ 1) in OGD/R versus control, including 626 upregulated and 502 downregulated genes. In OGD/R+tsRNA-3025a inhibitor versus OGD/R, 84 genes met the significance threshold (|log2FC| ≥ 1), 82 were upregulated, and 2 were downregulated (Figure S5A). Bioinformatic assessment using GSEA highlighted a marked concentration of transcripts involved in phosphatidylinositol signaling and autophagy in response to tsRNA-3025a knockdown (Figure S5B). tsRNAs have been reported to regulate gene expression by binding the 3′UTR of mRNAs in an AGO-dependent manner [27], and we hypothesized that tsRNA-3025a downregulates key protective genes during OGD/R. Using a Venn-diagram approach, we intersected RNAhybrid predictions, genes upregulated in OGD/R+tsRNA-3025a inhibitor versus OGD/R, and genes downregulated in OGD/R versus control, yielding eight overlapping candidates; among these, PIK3C2A drew our attention, showing marked upregulation in the RNA-seq dataset (Figure 7B). PIK3C2A encodes PI3K-c2α, which has distinct cellular roles, including regulation of receptor endocytosis linked to angiogenesis, cell migration, and autophagy [28]. Dual-luciferase assays confirmed that tsRNA-3025a targets the PIK3C2A 3′UTR at nucleotides 1365–1382 (Figure 7C). In AC16 cells, transient overexpression of tsRNA-3025a reduced PIK3C2A at both mRNA (Figure S5C) and protein levels (Figure 7D), whereas knockdown increased its expression. Consistent with these findings, Western blotting showed that OGD/R decreased PI3K-c2α and the LC3B-II/I ratio while increasing p62 expression (sequestosome 1/SQSTM1). In addition, overexpression of tsRNA-3025a further decreased PI3K-c2α and LC3B-II/I and increased p62, whereas the knockdown of tsRNA-3025a produced the opposite changes (Figure 7E).

To assess autophagic flux, we employed the tandem RFP-GFP-LC3 reporter. In this assay, red-only (RFP^+^/GFP^−^) puncta indicate autolysosomes and intact flux, whereas retention of yellow (RFP^+^/GFP^+^) puncta or a loss of puncta indicates impaired initiation and/or blocked flux. In the control group, abundant LC3 puncta were observed, consistent with active autophagy. In OGD/R-treated AC16 cells, both RFP-LC3 and GFP-LC3 puncta were markedly reduced, suggesting suppression of autophagosome formation. Overexpression of tsRNA-3025a further reduced LC3 puncta and overall RFP/GFP signal intensity, indicating aggravated inhibition of autophagy initiation and flux, whereas tsRNA-3025a knockdown improved the autophagic readouts under OGD/R (Figure 7F).

## 4. Discussion

In this study, we primarily unveil the dynamic landscape of I/R-responsive tsRNAs and identify tsRNA-3025a as a critical pathogenic driver and a prognostic biomarker for AMI. Initially, we report that reperfusion injury reshapes the cardiac small non-coding RNA profile, featuring a pronounced accumulation of tsRNAs. Among these, tsRNA-3025a displays cardiac-enriched expression and is specifically upregulated across murine I/R hearts, OGD/R-treated AC16 cells, and the plasma in ACS patients. Moreover, plasma tsRNA-3025a increases in AMI and UA patients and peaks rapidly post-reperfusion. The combination of tsRNA-3025, NT-proBNP and TnT shows superior sensitivity for predicting adverse post-infarction outcomes compared to single traditional biomarkers alone. Functionally, we provide evidence spanning from in vitro models to in vivo experiments, where tsRNA-3025a exacerbates cardiomyocyte apoptosis, mitochondrial dysfunction, cardiomyocytes proliferation and long-term cardiac fibrosis. Mechanistically, we delineate a novel axis wherein tsRNA-3025a suppresses the initiation and flux of protective autophagy by directly targeting PIK3C2A.

Myocardial I/R injury represents a frequent and debilitating consequence following revascularization therapy [29]. Because this pathology involves complicated molecular mechanisms and conventional cardiac injury biomarkers often lack robust predictive power for occurrence of reperfusion injury and adverse outcomes, the translation of effective drugs or biomarkers into clinical practice has remained challenging [30]. However, non-coding RNAs (ncRNAs) are increasingly recognized as potent regulators of disease pathogenesis and promising indicators across organ systems [31]. Once considered random degradation products, tsRNAs are now appreciated as functional ncRNAs with substantial therapeutic and biomarker potential [15]. For instance, a recent clinical study utilizing Pandora-seq identified tRF-Gly-GCC-06 as being closely associated with ACS stages and regulating macrophage pro-inflammatory activity [32]. Interestingly, we similarly observed stage-graded increases of tsRNA-3025a, which were modest in UA but peaked remarkably after reperfusion in AMI patients. In contrast to established biomarkers that typically rise post-infarction, tsRNA-3025a levels increase early during the unstable angina phase, anticipating the onset of AMI. This rapid response reflects its sensitivity to ischemic/hypoxic stress and positions it as a promising early-warning indicator for high-risk coronary artery disease [7]. Consistent with this advantage, plasma tsRNA-3025a significantly correlated with both troponin elevation and LVEF reduction in AMI. Crucially, incorporating tsRNA-3025a into a multi-marker model markedly improved the discrimination (AUC 0.793) for acute post-reperfusion heart failure, and elevated baseline levels conferred a 2.174-fold increased risk of MACE. Furthermore, patients with high tsRNA-3025a expression exhibited more severe reperfusion injury features and a higher burden of coronary risk factors, such as elevated LDL-c. Collectively, these findings suggest that tsRNA-3025a serves as a comprehensive stress sensor reflecting cardiac injury severity. Although further validation in larger cohorts is warranted, our data indicate that tsRNA-3025a offers complementary value to established markers, potentially optimizing risk stratification in ACS management.

Beyond its promising prognostic utility, our study highlights tsRNA-3025a as a potential therapeutic target. In the broader context of cardiovascular disease, tsRNAs are increasingly implicated in pathological progression. For example, tRF-Glu-CTC-013 has been proposed as both a marker and target in cardiac hypertrophy [33], while tiRNA-Met-CAT-002 has been shown to exacerbate myocardial injury in reperfusion models [21]. Our data expand this evolving field by demonstrating that tsRNA-3025a not only exhibits elevated levels in circulation but directly contributes to I/R injury pathogenesis. Notably, tsRNA-3025a overexpression exacerbated mitochondrial depolarization and apoptosis in vitro. Underlying these phenotypes is the disruption of cellular redox homeostasis. Classical antioxidant defense systems, such as the Nrf2/Keap1 axis, play a crucial role in neutralizing the excessive reactive oxygen species (ROS) generated during I/R. However, the severe autophagic blockade induced by tsRNA-3025a prevents the clearance of dysfunctional mitochondria, leading to a catastrophic ROS burst. This overwhelming oxidative stress likely exhausts and secondarily impairs vital antioxidant enzyme pathways, profoundly exacerbating the acute cellular damage. More importantly, extending our observation to the chronic phase (28 days post-I/R), we found that inhibiting tsRNA-3025a significantly attenuated cardiac fibrosis and preserved ventricular geometry. This is particularly pivotal, as adverse remodeling is the primary driver of post-infarction heart failure [34]. The ability of tsRNA-3025a inhibition to confer long-term cardio-protection suggests that it acts early in the reperfusion injury to limit the initial infarct size and reduces the fibrotic replacement.

Mechanistically, we identified tsRNA-3025a as a 3′-tRF derived from tRNA-Ser. While prior work has emphasized tiRNAs linked to cellular stress and translational control [13], 3′-tRFs remain comparatively under-studied, though they have been implicated in the tumorigenesis and prognosis of cancers [35,36]. tiRNAs often suppress translation via competitive binding to translation-related proteins [19,37], whereas tRFs more frequently act in an AGO-dependent, miRNA-like manner through sequence complementarity to target 3′UTR [27]. In our study, RNA-sequencing and GSEA indicated a specific enrichment in autophagy and phosphatidylinositol signaling pathways following tsRNA-3025a knockdown. We identified PIK3C2A as a putative target of tsRNA-3025a by integrating bioinformatic predictions with transcriptome analysis of differentially expressed genes. This gene encodes PI3K-c2α that catalyzes phosphatidylinositol-3-phosphate and phosphatidylinositol 3,4-bisphosphate synthesis, thereby regulating multiple cellular processes including vesicular trafficking, receptor endocytosis and cell migration [28]. Significantly, Merrill et al. confirmed that PI3K-c2a knockdown suppresses the maturation of autophagosomes and endocytic vesicles, revealing PI3K-c2a as an essential positive regulator of autophagy [38]. Crucially, our experimental data demonstrate that tsRNA-3025a directly binds the 3′UTR of PIK3C2A, causing marked downregulation of PI3K-c2α protein. This post-transcriptional silencing mechanism implies critical pathological roles. Recent studies show that the reduced level of PIK3C2A is associated with CAD pathogenesis and ACS prognosis [39,40]. We therefore propose that tsRNA-3025a-mediated suppression of PI3K-c2α impairs autophagy in cardiomyocytes, constituting a key mechanism underlying I/R injury.

It is widely acknowledged that the relationship between autophagy and I/R injury is highly context-dependent and remains a subject of debate [41]. On one hand, some studies report that OGD/R suppresses autophagy [22,42,43], while others suggest it promotes autophagic activity [44,45]. Furthermore, controversies exist regarding whether autophagy is beneficial or detrimental. For instance, upregulation of SLC39A7 or RIPK3 during reperfusion has been shown to inhibit protective mitophagy, thereby promoting apoptosis [46]. Conversely, ALDH2-mediated inhibition of constitutive PINK1-PRKN mitophagy unexpectedly alleviated I/R injury [47]. We propose that the outcome likely depends on the dynamic balance between flux initiation and lysosomal clearance. In our experiment, we observed a clear blockage of autophagic flux (accumulation of p62 and LC3B-II) under OGD/R conditions. Crucially, tsRNA-3025a aggravates the blockage of autophagic flux and inhibits the autophagy initiation. By repressing PIK3C2A, tsRNA-3025a inhibited the maturation of autophagosomes, leading to the accumulation of damaged mitochondria and subsequent cell death. Conversely, restoring PIK3C2A levels via tsRNA-3025a knockdown re-engaged protective autophagy flux, and promoted cell survival. Thus, our findings position the tsRNA-3025a/PIK3C2A axis at a critical decision point between cell survival and death during hypoxia stress.

In summary, this study identifies tsRNA-3025a as a specific, stress-induced regulator of myocardial I/R injury. We demonstrate its utility as a sensitive prognostic biomarker for AMI patients and as a molecular driver of I/R injury that suppresses PIK3C2A-dependent protective autophagy.

However, there are still some limitations in this study. First, while our prospective cohort provides valuable data, the sample size is relatively small, and the causal clinical relationship between tsRNA-3025a and adverse AMI outcomes requires validation in large-scale, multi-center trials. Second, tsRNA-seq was performed on whole heart tissue; although tsRNA-3025a increases in cardiomyocytes, the potential contributions from other cell types, such as infiltrating immune cells, remain to be delineated. Finally, while our data strongly support the tsRNA-3025a/PIK3C2A/autophagy axis, definitive in vivo rescue experiments would be needed to strictly establish causality.

## 5. Conclusions

This study identifies tsRNAs enriched in myocardial I/R injury and highlights tsRNA-3025a as a promising biomarker for predicting cardiac injury and adverse events in AMI. tsRNA-3025a exacerbates apoptosis, promotes mitochondrial membrane depolarization, impairs cardiomyocyte proliferation and repair, and reduces myocardial salvage under I/R stress. Mechanistically, tsRNA-3025a targets PIK3C2A and suppresses autophagy. These findings refine our understanding of reperfusion injury and point toward tsRNA-3025a/PIK3C2A–autophagy as a potential axis for biomarker development and therapeutic intervention.

## Figures and Tables

**Table 1:** Demographic and clinical features.

Characteristics	Controls (*n* = 63)	AMI (*n* = 80)	*p* Value
Gender, n (%)			
Female	29 (46.0)	16 (20.0)	
Male	34 (54.0)	64 (80.0)	0.001
Age, years	60 (52, 60)	63 (52, 73)	0.079
Weight, kg	67 (62, 75)	70 (64, 80)	0.066
Height, cm	168 (160, 172)	170 (165, 175)	0.052
Risk Factor, n (%)			
HBP	11 (22.40)	52 (65.0)	<0.001
DM	3 (6.10)	30 (37.50)	<0.001
Hyperlipemia	9 (18.40)	29 (36.30)	<0.001
Smoke	10 (20.40)	48 (60.0)	<0.001
Culprit vessel, n (%)			
LA	/	59 (73.7)	
RA	/	21 (26.3)	/
Onset to door time, hours	/	6 (3, 12.5)	/
TIMI classification before PCI, n (%)			
0–1	/	70 (87.5)	
2–3	/	10 (12.5)	/
Echocardiography			
LVEF, %	62 (60, 63)	55 (46, 59)	<0.001
LA, mm	37 (34, 39)	37 (35, 40)	0.047
Lab			
WBC, 10^9^/L	6.14 (5.07, 6.84)	9.62 (7.82, 12.39)	<0.001
Hb, g/L	139 (129, 148)	146 (136, 157)	0.041
CK-MB, U/L	13.0 (10.0, 15.80) ^b^	37.55 (16.25, 192.50)	0.001
cTnT, ng/L	7.50 (4.85, 10.78) ^a^	210 (40.08, 1162.50)	<0.001
NT-proBNP, pg/mL	59 (27, 114) ^b^	251 (85, 937)	0.003
TC, mmol/L	3.77 (3.18, 5.08) ^c^	4.42 (3.80, 5.51)	0.020
TG, mmol/L	1.27 (0.86, 1.68) ^c^	1.70 (1.10, 2.33)	0.082
LDL-C, mmol/L	2.40 (1.77, 3.25) ^c^	3.24 (2.58, 4.02)	<0.001
ALT, U/L	17.80 (14.38, 34.23)	27.50 (14.75, 43.50)	0.176
AST, U/L	20.0 (17.30, 24.0)	43.50 (19.0, 95.25)	0.167
SCr, μmol/L	65.70 (50.10, 77.20)	78.15 (63.30, 91.23)	0.003
tsRNA-3025a, −ΔCT	−0.91 (−2.24, 2.32)	4.02 (3.03, 5.99)	<0.001
Treatment, n (%)			
Aspirin	39 (61.9)	80 (100)	<0.001
Clopidogrel	12 (19.0)	2 (2.50)	<0.001
Ticagrelor	0 (0)	78 (97.50)	<0.001
CCB	9 (14.3)	14 (17.70)	<0.001
ARNI	3 (4.80)	33 (41.30)	<0.001
ACEI/ARB	7 (14.30)	20 (25.0)	<0.001
Statin	20 (31.70)	80 (100)	<0.001
PCSK9i	1 (1.60)	55 (68.80)	<0.001

Data are presented as n (%) or median (quartile). ^a^ available in 53 patients. ^b^ available in 39 patients. ^c^ available in 59 patients. Abbreviations: HBP, high blood pressure; DM, diabetes mellitus; LA, left coronary artery; RA, right coronary artery; TIMI, thrombolysis in myocardial infarction; LVEF, left ventricular ejection fraction; LA, left atrium; CRP, C-reactive protein; WBC, white blood cell; Hb, hemoglobin; TnT, troponin T; TG, triglyceride; TC, cholesterol; LDL-C, low-density lipoprotein; ALT, alanine aminotransferase; AST, aspartate aminotransferase; Scr, serum creatinine; CK, creatine kinase; NT-proBNP, N-terminal B-type natriuretic peptide; CCB, calcium channel blocker; ARNI, angiotensin receptor-neprilysin inhibitor; PCSK9i, proprotein convertase subtilisin/kexin type 9 inhibitor.

**Table 2:** Comparison between high and low expression of tsRNA-3025a in AMI.

Characteristics	High Expression (*n* = 20)	Low Expression (*n* = 60)	*p* Value
Gender, n (%)			
Female	4	12	
Male	16	48	1.000
Age, years	59 (49, 71)	65 (56, 75)	0.034
Height, kg	170 (160, 175)	170 (165, 175)	0.947
Weight, cm	72 (61, 81)	70 (63, 80)	0.493
Risk Factor, n (%)			
HBP	10 (50)	42 (70)	0.115
DM	8 (40)	22 (37)	0.796
Hyperlipemia	11 (55)	18 (30)	0.044
Smoke	14 (70)	34 (57)	0.292
Killip grade, n (%)			
0–1	14 (70.0)	52 (86.60)	
2–4	6 (30.0)	8 (13.40)	0.089
Onset to door time, hours	7.0 (2.25, 10.0)	6.0 (3.80, 13.0)	0.463
TIMI classification before PCI, n (%)			
0–1	18 (90.0)	52 (86.7)	
2–3	2 (10.0)	8 (13.3)	0.696
Echocardiography			
LVEF, %	50 (40, 58)	55 (50, 59)	0.012
LA, mm	37 (35, 40)	37 (35, 40)	0.298
Lab			
WBC, 10^9^/L	11.51 (7.89, 15.01)	9.40 (7.82, 11.87)	0.119
Hb, g/L	148 (136, 158)	145 (136, 157)	0.861
CK-MB, U/L	33 (15, 270)	50 (17, 166)	0.673
cTnT, ng/L	571.50 (37.10, 3517.0)	153.30 (40.20, 900.20)	0.045
NT-proBNP, pg/mL	291 (108, 796)	250 (81, 959)	0.381
TC, mmol/L	5.12 (3.67, 5.92)	4.38 (3.83, 5.37)	0.514
TG, mmol/L	2.05 (1.47, 3.74)	1.48 (1.05, 2.19)	0.004
LDL-C, mmol/L	3.87 (2.80, 4.56)	3.17 (2.42, 3.82)	0.030
Treatment, n (%)			
Aspirin	20 (100)	60 (100)	/
Ticagrelor	19 (95.0)	59 (98.30)	0.408
ARNI	7 (35.0)	26 (43.30)	0.512
ACEI/ARB	6 (30.0)	14 (23.30)	0.551
CCB	2 (10.0)	12 (20.30)	0.499
Statin	20 (100)	60 (100)	/
PCSK9i	13 (65.0)	42 (70.0)	0.676

Data are presented as n (%) or median (quartile). Abbreviations: HBP, high blood pressure; DM, diabetes mellitus; LA, left coronary artery; RA, right coronary artery; TIMI, thrombolysis in myocardial infarction; LVEF, left ventricular ejection fraction; LA, left atrium; CRP, C-reactive protein; WBC, white blood cell; Hb, hemoglobin; TnT, troponin T; LDL-C, low-density lipoprotein; ALT, alanine aminotransferase; AST, aspartate aminotransferase; Scr, serum creatinine; CK, creatine kinase; NT-proBNP, N-terminal B-type natriuretic peptide; CCB, calcium channel blocker; ARNI, angiotensin receptor-neprilysin inhibitor; PCSK9i, proprotein convertase subtilisin/kexin type 9 inhibitor.

## Data Availability

The original contributions presented in this study are included in the article/Supplementary Material. Further inquiries can be directed to the corresponding authors.

## Supplementary Materials

The following supporting information can be downloaded at https://www.mdpi.com/article/10.3390/jcdd13060266/s1, Figure S1: Validation of myocardial ischemia/reperfusion injury mouse model. (A) Representative echocardiographic images from sham and I/R groups. LVEF, LVFS, LVEDD and heart rate were measured in sham (n = 6) and I/R (n = 8) group. (B) TUNEL staining were conducted to assess myocardial apoptosis (Scale bar = 50 μm). (C) HE staining shows pathological damage in I/R injury (Scale bar = 50 μm). Data are expressed as mean ± SE. ** *p* < 0.01, *** *p* < 0.001, ns indicates not significant; Figure S2: Flow diagram for enrollment of participants; Figure S3: Subcellular localization and apoptotic regulation of tsRNA-3025a under OGD/R conditions. (A) Western blot analysis of apoptosis markers Bax, in control, OGD/R, OGD/R+inhibitor, and OGD/R+mimic groups. (B) RNA FISH analysis of tsRNA-3025a in control and OGD/R groups: red denotes tsRNA-3025a and blue denotes nuclei. (scale bar = 5 μm); Figure S4. tsRNA-3025a regulates cardiomyocyte apoptosis and regenerative proliferation following myocardial I/R injury. (A) TUNEL staining to assess apoptosis; quantification of TUNEL-positive cells in the ischem-ic/reperfused area in I/R group (n = 6) and sham group (n = 4, Scale bar = 50 µm). Arrows indicate the TUNEL^+^ cardiomyocyte. (B,C) Representative immunostaining images of Ki67^+^ or pH3^+^ (red) cardiomyocytes in I/R mice treated with tsRNA-3025a antagomir (n = 6 per group, Scale bar = 25 μm). Arrows indicate the Ki67^+^/pH3^+^ cardiomyocyte. Data are expressed as mean ± SE. * *p* < 0.05, ** *p* < 0.01, *** *p* < 0.001. Statistical significance uses one-way ANOVA with Tukey’s post-hoc test; Figure S5: tsRNA-3025a is involved in the autophagy pathway by regulating the expression of PIK3C2A. (A) Differential gene expression was analyzed via volcano plots for two contrasts: OGD/R+tsRNA inhibitor vs. OGD/R, and OGD/R vs. control. (B) GSEA showing enrichment relationships between tsRNA-3025a and phosphatidylinositol-related pathways or autophagy-related pathways. (C) tsRNA-3025a overexpression or knockdown in AC16 cells decrease or increase PIK3C2A expres-sion at the mRNA level. Data are expressed as mean ± SE. ** *p* < 0.01, **** *p* < 0.0001, ns indicates not significant. Statistical significance uses one-way ANOVA with Tukey’s post-hoc test; Table S1: Comparison between unstable angina and stable angina; Table S2: Univariable and multivariable risk analysis of elevated tsRNA-3025a in AMI patients; Table S3: Univariable and multivariable risk analysis of MACEs in hospital; Table S4: Primers used for real-time PCR assay; Table S5: Small interfering RNA (siRNA) sequences; Table S6: RNA oligonucleotide sequences; Table S7: RNA-seq analysis of antioxidant enzyme pathway genes.

## Abbreviations

The following abbreviations are used in this manuscript:

AAR	Area at risk
ACS	Acute coronary syndrome
Ago	Argonaute
AMI	Acute myocardial infarction
ANG	Angiogenin
AUC	Area under the curve
Bax	Bcl-2-associated X protein
CHD	Coronary heart disease
CI	Confidence interval
CK-MB	Creatine kinase-MB
cTnT	Cardiac troponin T
FISH	Fluorescence in situ hybridization
GSEA	Gene set enrichment analysis
H&E	Hematoxylin and eosin
I/R	Ischemia–reperfusion
LAD	Left anterior descending coronary artery
LDH	Lactate dehydrogenase
LVEDD	Left ventricular end-diastolic dimension
LVEF	Left ventricular ejection fraction
LVFS	Left ventricular fractional shortening
MACEs	Major adverse cardiovascular events
miRNA	microRNA
ncRNA	Non-coding RNA
NT-proBNP	N-terminal pro-brain natriuretic peptide
OGD/R	Oxygen-glucose deprivation/reoxygenation
OR	Odds ratio
PCI	Percutaneous coronary intervention
PI3K	Phosphatidylinositol 3-kinase
PIK3C2A	Phosphatidylinositol-4-phosphate 3-kinase catalytic subunit type 2 alpha
qRT-PCR	Quantitative real-time polymerase chain reaction
ROC	Receiver operating characteristic
TEM	Transmission electron microscopy
tiRNA	tRNA-derived stress-induced RNA
tRF	tRNA-derived fragment
tRNA	Transfer RNA
tsRNA	Transfer RNA-derived small RNA
TTC	2,3,5-Triphenyltetrazolium chloride
TUNEL	Terminal deoxynucleotidyl transferase dUTP nick end labeling
UA	Unstable angina
VT/VF	Ventricular tachycardia/ventricular fibrillation
