# Supplementary material for: tsRNA-3025a Impairs Mitochondrial Function and Autophagy to Inhibit Myocardial Regeneration and Repair Following Ischemia–Reperfusion Injury
Source: J Cardiovasc Dev Dis. 2026 Jun 12;13(6):266. doi: 10.3390/jcdd13060266 (PMC13302443; doi:10.3390/jcdd13060266)
Supplement: Supplementary file 1 [file jcdd-13-00266-s001.zip › Supplementary Material.pdf]

SUPPLEMENTARY MATERIAL

Supplementary figure

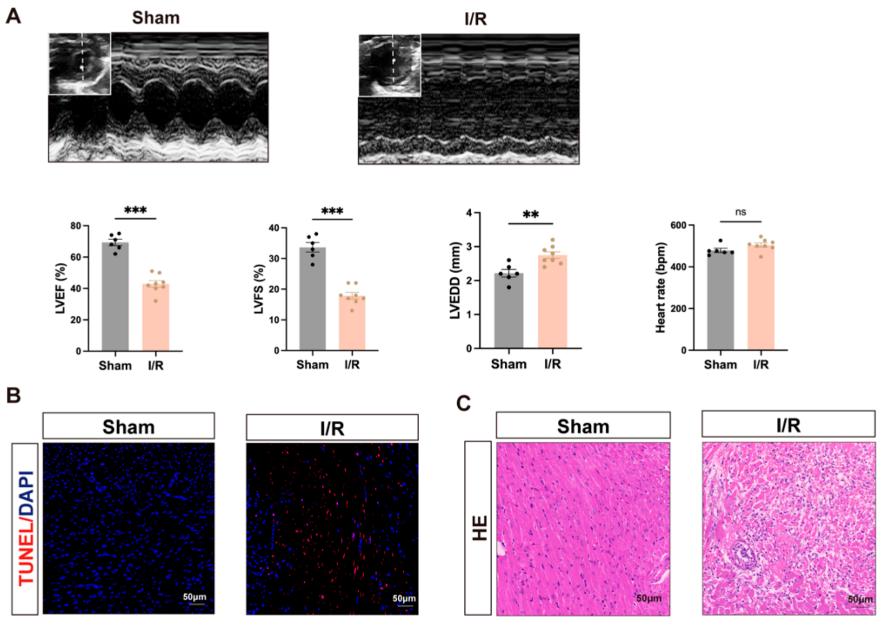

Figure S1. Validation of myocardial ischemia/reperfusion injury mouse model.

(**A**) Representative echocardiographic images from sham and I/R groups. LVEF, LVFS, LVEDD and heart rate were measured in sham (n=6) and I/R (n=8) group. (**B**) TUNEL staining were conducted to assess myocardial apoptosis (Scale bar=50μm). (**C**) HE staining shows pathological damage in I/R injury (Scale bar=50μm). Data are expressed as mean ± SE. **p<0.01, ***p<0.001, ns indicates not significant

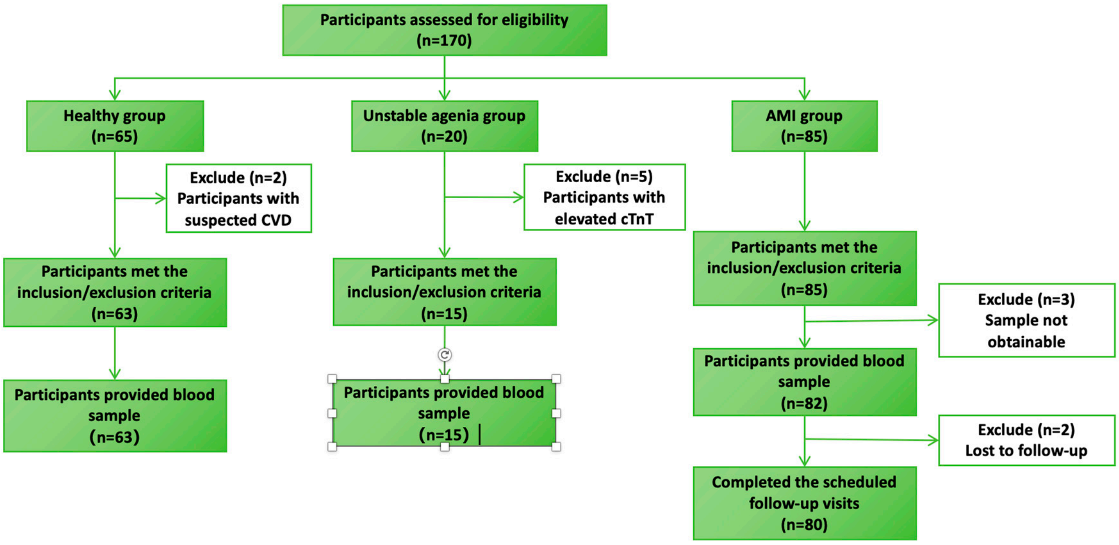

Figure S2. Flow diagram for enrollment of participants.

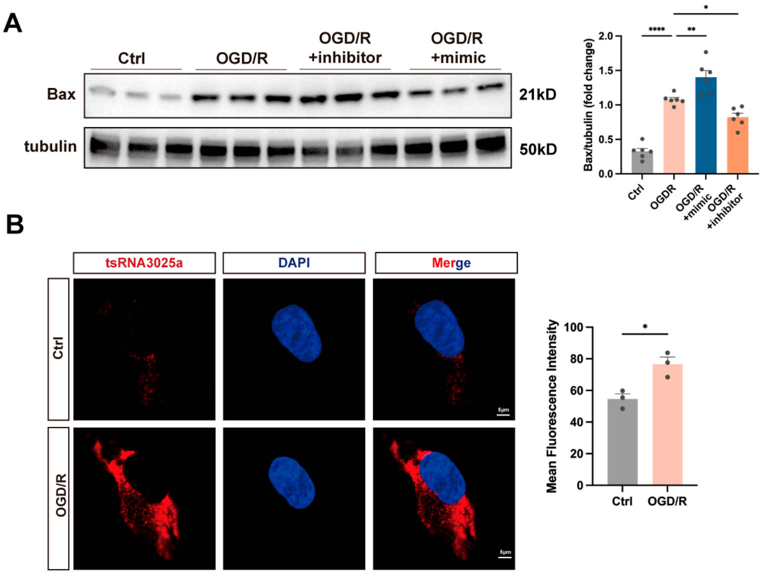

Figure S3. Subcellular localization and apoptotic regulation of tsRNA-3025a under OGD/R conditions.

(**A**) Western blot analysis of apoptosis markers Bax, in control, OGD/R, OGD/R+inhibitor, and OGD/R+mimic groups. (**B**) RNA FISH analysis of tsRNA-3025a in control and OGD/R groups: red denotes tsRNA-3025a and blue denotes nuclei. (scale bar = 5μm).

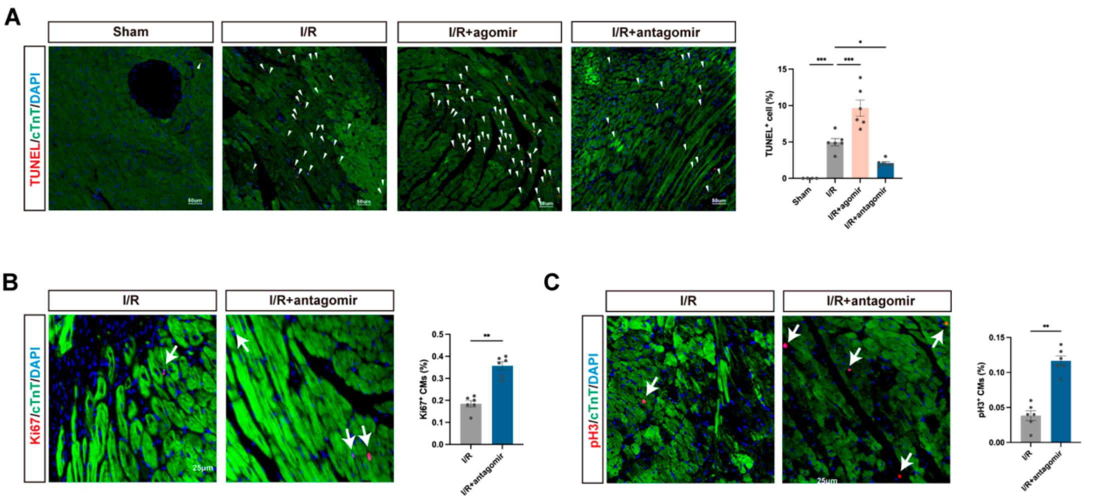

Figure S4. tsRNA-3025a regulates cardiomyocyte apoptosis and regenerative proliferation following myocardial I/R injury.

(**A**) TUNEL staining to assess apoptosis; quantification of TUNEL-positive cells in the ischemic/reperfused area in I/R group (n=6) and sham group (n=4, Scale bar=50µm). Arrows indicate the TUNEL^{+} cardiomyocyte. (**B-C**) Representative immunostaining images of Ki67^{+} or pH3^{+} (red) cardiomyocytes in I/R mice treated with tsRNA-3025a antagomir (n=6 per group, Scale bar=25µm). Arrows indicate the Ki67^{+}/pH3^{+} cardiomyocyte. Data are expressed as mean ± SE. *p<0.05, **p<0.01, ***p<0.001. Statistical significance uses one-way ANOVA with Tukey's post-hoc test.

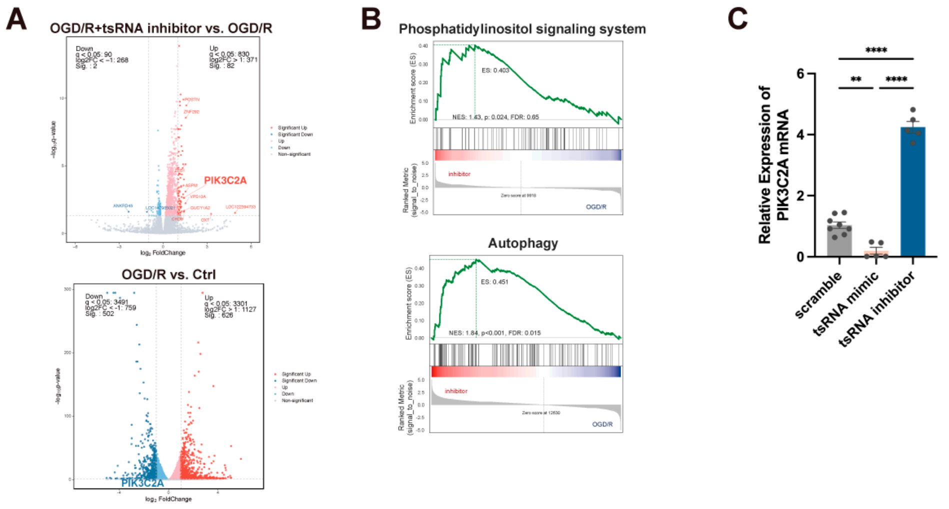

Figure S5. tsRNA-3025a is involved in the autophagy pathway by regulating the expression of PIK3C2A.

(**A**) Differential gene expression was analyzed via volcano plots for two contrasts: OGD/R+tsRNA inhibitor vs. OGD/R, and OGD/R vs. control. (**B**) GSEA showing enrichment relationships between tsRNA-3025a and phosphatidylinositol-related pathways or autophagy-related pathways. (**C**) tsRNA-3025a overexpression or knockdown in AC16 cells decrease or increase PIK3C2A expression at the mRNA level. Data are expressed as mean ± SE. ****p*<0.01, ******p*<0.0001, ns indicates not significant. Statistical significance uses one-way ANOVA with Tukey's post-hoc test.

Supplementary table

Table S1. Comparison between unstable angina and stable angina

**Characteristics**	**Stable angina (n=15)**	**Unstable angina (n=15)**	***P* value**
Gender, n (%)			
Female	5 (33.3)	4 (26.7)	
Male	10 (66.7)	11 (73.3)	0.690
Age, years	69 (65, 73)	70 (62, 75)	0.880
Height, kg	168 (160, 171)	166 (163, 172)	0.385
Weight, cm	70 (66, 75)	70 (66, 75)	0.807
Risk Factor, n (%)			
HBP	11 (73.3)	9 (60.0)	0.700
DM	4 (26.7)	7 (46.7)	0.256
Hyperlipemia	2 (13.3)	4 (26.7)	0.651
Smoke	5 (33.3)	9 (60.0)	0.143
Echocardiography			
LVEF, %	62 (60, 63)	62 (60, 63)	0.984
LA, mm	37 (35, 39)	39 (34, 40)	0.845
Lab			
WBC, 10^9/L	6.46 (4.54, 6.75)	5.78 (4.74, 8.02)	0.321
Hb, g/L	133 (129, 145)	138 (134, 148)	0.661
CK-MB, U/L	15.6 (11.6, 16.65)	15.9 (13.5, 17.6)	0.497
cTnT, ng/L	10.3 (5.4, 11.4)	10.6 (8.7, 13.9)	0.280
NT-proBNP, pg/mL	67 (35, 122)	42 (26, 103)	0.990
TC, mmol/L	3.41 (2.83, 4.00)	4.00 (2.79, 4.51)	0.216
TG, mmol/L	0.97 (0.77, 1.42)	1.26 (0.89, 1.92)	0.113
LDL-C, mmol/L	1.58 (1.35, 2.15)	2.44 (1.52, 2.54)	0.076
tsRNA-3025a, -ΔCT	1.64 (-1.69, 2.17)	2.4 (1.40, 3.84)	**0.022**
Treatment, n (%)			
Aspirn	10 (66.7)	12 (80.0)	0.682
ARNI	1 (6.7)	2 (13.3)	1.000
ACEI/ARB	6 (40.0)	2 (13.3)	0.215
CCB	4 (26.7)	3 (20.0)	1.000
Statin	13 (86.7)	12 (80.0)	1.000
PCSK9i	0 (0)	2 (13.3)	0.483

Data are presented as n (%) or median (quartile).

Abbreviations: HBP, high blood pressure; DM, diabetes mellitus; LA, left coronary artery; RA, right coronary artery; TIMI, thrombolysis in myocardial infarction; LVEF, left ventricular ejection fraction; LA, left atrium; CRP, C-reactive protein; WBC, white blood cell; Hb, hemoglobin; cTnT, troponin T; TG, triglyceride; TC, cholesterol; LDL-C, low-density lipoprotein; ALT, alanine aminotransferase; AST,

aspartate aminotransferase; Scr, serum creatinine; CK-MB, creatine kinase-MB; NT-proBNP, N-terminal B-type natriuretic peptide; CCB, calcium channel blocker; ARNI, angiotensin receptor-neprilysin inhibitor; PCSK9i, proprotein convertase subtilisin/kexin

Table S2. Univariable and multivariable risk analysis of elevated tsRNA-3025a in AMI patients

**Variable**	**Univariate**		**Multivariate**	
	p-value	OR (95%CI)	*p-value*	OR (95%CI)
Age	0.040	0.958 (0.920, 0.998)	0.042	0.945 (0.896, 0.998)
Smoke	0.295	1.784 (0.603, 5.276)		
Killip grade	0.066	1.583 (0.971, 2.582)	0.029	2.058 (1.076, 3.936)
SCr	0.744	1.003 (0.984, 1.023)		
cTnT	0.150	1.205 (0.935, 1.553)		
TG	0.009	1.839 (1.166, 2.901)	0.002	2.682 (1.436, 5.012)
LDL	0.035	1.816 (1.043, 3.163)		
LVEF	0.016	0.925 (0.868, 0.986)	0.023	0.890 (0.805, 0.984)

Abbreviations: cTnT, troponin T; TG, triglyceride; LDL-C, low-density lipoprotein; Scr, serum creatinine; LVEF, left ventricular ejection fraction.

Table S3. Univariable and multivariable risk analysis of MACEs in hospital

**Variable**	**Univariate**		**Multivariate**	
	p-value	OR (95%CI)	*p-value*	OR (95%CI)
Age	0.211	1.049 (0.973, 1.130)	0.047	1.115 (1.001,1.243)
Smoke	0.522	1.744 (0.317, 9.594)		
TIMI grade	0.386	0.386 (0.098, 1.520)		
SCr	0.100	1.021 (0.996, 1.046)		
cTnT	0.974	1.007 (0.656, 1.547)		
TG	0.551	0.776 (0.338, 1.782)		
LVEF	0.026	0.896 (0.813, 0.987)	0.023	0.885 (0.797, 0.983)
tsRNA-3025a	0.018	1.669 (1.092, 2.553)	0.002	2.174 (1.238, 3.816)

Abbreviations: TIMI, thrombolysis in myocardial infarction; TnT, troponin T; TG, triglyceride; Scr, serum creatinine; LVEF, left ventricular ejection fraction.

Table S4. Primers used for real-time PCR assay

Gene	Forward (5'-3')	Reverse (5'-3')
**Poly(A)-tailing**		
Universal reverse mQ		provided by manufacturer (Sangon)
tsRNA-3019a-F	TTATATTATCCCAGCGGGGCCTC	
tsRNA-3003a-F	ATAATATCCCGGACGAGCCCC	
tsRNA-3025a-F	ATCCTGCCGACTACGCCA	
tsRNA-3038a-F	TTATATCCCCGTACGGGCCAC	
tsRNA-3035a-F	AATATGTCCCTTCGTGGTCGCCA	
**Stem-loop**		
Universal reverse mQ		ATCCAGTGCAGGGTCCGAGG
tsRNA-3003a-F	AATTGCTCATCCCGGACGAGC	
tsRNA-3003a-RT	GTCGTATCCAGTGCAGGGTCCGAG GTATTCGCACTGGATACGACTGGG GG	
tsRNA-3038a-F	CGCGTCCCCGTACGGG	
tsRNA-3038a-RT	GTCGTATCCAGTGCAGGGTCCGAG GTATTCGCACTGGATACGACTGGT GG	
tsrna-3025a-F	AAGCGCCTATCCTGCCGACTA	
tsrna-3025a-RT	GTCGTATCCAGTGCAGGGTCCGAG GTATTCGCACTGGATACGACTGGC GT	
tsrna-3019a-F	CGCGATCCCAGCGGGG	
tsrna-3019a-RT	GTCGTATCCAGTGCAGGGTCCGAG GTATTCGCACTGGATACGACTGGA GG	
**mRNA**		
U6#1	CGATACAGAGAAGATTAGCATGG C	AACGCTTCACGAATTTGCGT
U6#2	provided by manufacturer (Vazyme)	provided by manufacturer (Vazyme)
β-actin	CACCATTGGCAATGAGCGGTTC	AGGTCTTTGCGGATGTCCAC GT
GAPDH	GCATTGCCCTCAACGACCAC	CCACCACCCTGTTGCTGTAG

Tables S5. Small interfering RNA (siRNA) sequences

Gene	Sense (5'-3')	Antisense (5'-3')
NC siRNA	UUCUCCGAACGUGUCACGUTT	ACGUGACACGUUCGGAGAATT
PIK3C2A siRNA#1	GUCUGAGGAUAUCAGUAAA/dT//dT/	UUUACUGAUAUCCUCAGAC/dT//dT/
PIK3C2A siRNA#2	GAGUUGUCAAGCAGCACCA/dT//dT/	UGGUGCUGCUUGACAACUC/dT//dT/
PIK3C2A siRNA#3	GGUUUAUAACAAGCAGGAU/dT//dT/	AUCCUGCUUGUUAUAAACC/dT//dT/

Tables S6. RNA oligonucleotide sequences

Gene	Sequence (5'-3')	Modification
tsRNA-3025a mimic-sense	AUCCUGCCGACUACGCCA	2`-O-Methyl
tsRNA-3025a mimic-antisense	GCGUAGUCGGCAGGAUUU	2`-O-Methyl
tsRNA-3025a inhibitor	/i2OMeU//i2OMeG//i2OMeG//i2OMeC//i2OMeG//i2OMeU//i2OMeA//i2OMeG//i2OMeU//i2OMeC//i2OMeG//i2OMeG//i2OMeC//i2OMeA//i2OMeG//i2OMeG//i2OMeA//i2OMeU/	2`-O-Methyl
NC mimic	UUGUACUACACAAAAGUACUG	2`-O-Methyl
NC inhibitor	CAGUACUUUUGUGUAGUACAA	2`-O-Methyl
tsRNA-3025a antagomir	/i2OMeU/*/i2OMeG/*/i2OMeG//i2OMeC//i2OMeG//i2OMeU//i2OMeA//i2OMeG//i2OMeU//i2OMeC//i2OMeG//i2OMeG//i2OMeC//i2OMeA//i2OMeG/*/i2OMeG/*/i2OMeA/*/i2OMeU/*	3`Cholesteryl0.0<br>Phosphorothioate72<br>2`-O-Methyl-U360<br>2`-O-Methyl-G720<br>2`-O-Methyl-C270
tsRNA-3025a agomir -Sense	AUCCUGCCGACUACGCCA	
tsRNA-3025a agomir -Antisense	/i2OMeG/*/i2OMeC/*/i2OMeG//i2OMeU//i2OMeA//i2OMeG//i2OMeU//i2OMeC//i2OMeG//i2OMeG//i2OMeC//i2OMeA//i2OMeG//i2OMeG//i2OMeA/*/i2OMeU/*/i2OMeU/*/i2OMeU/*	3`Cholesteryl0.0<br>Phosphorothioate24<br>2`-O-Methyl-U75<br>2`-O-Methyl-G105<br>2`-O-Methyl-C45<br>2`-O-Methyl-A45
NC antagomir	/i2OMeC/*/i2OMeA/*/i2OMeG//i2OMeU//i2OMeA//i2OMeC//i2OMeU//i2OMeU//i2OMeU//i2OMeU//i2OMeG//i2OMeU//i2OMeG//i2OMeU/i2OMeA//i2OMeG//i2OMeU//i2OMeA//i2OMeC/*/i2OMeA/*/i2OMeA/*/i2OMeA/*	3`Cholesteryl0.0<br>Phosphorothioate72<br>2`-O-Methyl-U360<br>2`-O-Methyl-G720<br>2`-O-Methyl-C270

Scramble agomir -Sense	CCAUUCGCACCCUAAGCC	
Scramble agomir -Antisense	/i2OMeG/*/i2OMeG/*/i2OMeC//i2OMeU//i2OMeU//i2OMeA//i2OMeG//i2OMeG//i2OMeG//i2OMeU//i2OMeG//i2OMeC//i2OMeG//i2OMeA//i2OMeA//i2OMeU//i2OMeG/*/i2OMeG/*/i2OMeU/*/i2OMeU/*	3`Cholesteryl0.0<br>Phosphorothioate24<br>2`-O-Methyl-U75<br>2`-O-Methyl-G105<br>2`-O-Methyl-C45<br>2`-O-Methyl-A45
